# The Role of CYP3A in Health and Disease

**DOI:** 10.3390/biomedicines10112686

**Published:** 2022-10-24

**Authors:** Lyubov S. Klyushova, Maria L. Perepechaeva, Alevtina Y. Grishanova

**Affiliations:** Institute of Molecular Biology and Biophysics, Federal Research Center of Fundamental and Translational Medicine, Timakova Str. 2, 630117 Novosibirsk, Russia

**Keywords:** CYP3A, CYP3A regulation, CYP3A endogenous substrates, drug-metabolizing enzymes, CYP3A in pathology

## Abstract

CYP3A is an enzyme subfamily in the cytochrome P450 (CYP) superfamily and includes isoforms CYP3A4, CYP3A5, CYP3A7, and CYP3A43. CYP3A enzymes are indiscriminate toward substrates and are unique in that these enzymes metabolize both endogenous compounds and diverse xenobiotics (including drugs); almost the only common characteristic of these compounds is lipophilicity and a relatively large molecular weight. CYP3A enzymes are widely expressed in human organs and tissues, and consequences of these enzymes’ activities play a major role both in normal regulation of physiological levels of endogenous compounds and in various pathological conditions. This review addresses these aspects of regulation of CYP3A enzymes under physiological conditions and their involvement in the initiation and progression of diseases.

## 1. Introduction

The CYP3A subfamily is affiliated with the cytochrome P450 (CYP) superfamily, which represents monooxygenases that catalyze the breakdown of various substances via hydroxylation and epoxidation with the participation of an electron donor (NADPH) and molecular oxygen [1]. CYP enzymes function as the first line of defense against exogenous chemical agents [2]. CYP enzymes are responsible for approximately three-quarters of all drug metabolism reactions in the human body [3,4]. CYP enzymes are involved in many critical metabolic reactions, including the metabolism of steroid hormones, bile acids, polyunsaturated fatty acids, leukotrienes, and eicosanoids [3].

Genes of CYP enzymes have been found in the genetic material of representatives of all kingdoms of living organisms, including plants. There are 57 known functional *CYP* genes in the human genome, aside from 58 pseudogenes whose protein products are enzymes metabolizing a wide range of endogenous and exogenous chemical compounds [2,5,6]. The genes of CYP enzymes are categorized into 18 families and 43 subfamilies based on the percentage of amino acid sequence homology. Just 3 families—CYP2, CYP3, and CYP4—contain more genes than the other 15 families combined [3,7]. The human CYP3 family consists of a single subfamily, CYP3A, which contains four genes (*CYP3A4*, *CYP3A5*, *CYP3A7*, and *CYP3A43*) encoding four functional enzymes [5,6,8,9,10]. 

CYP3A is a major subfamily in the cytochrome P450 superfamily. CYP3A enzymes are involved in the metabolism of more than 30% [11] and according to other reports 45–60% [12,13] of all pharmaceutical drugs currently on the market. CYP3A enzymes also metabolize some endogenous substrates, including hormones and bile acids, as well as nonpharmaceutical xenobiotics [11,12,14]. 

Expression of CYP3A enzymes is regulated and varies under the influence of various exogenous (drugs, chemicals, and diets) and endogenous factors (fatty acids, hormones, cytokines, and microRNAs [miRs or miRNAs]) [11].

CYP3A enzymes’ activity can be influenced by anthropogenic environmental chemicals: organophosphates, carbamates, parabens, benzotriazole UV stabilizers, and plasticizers [11,12]. Natural compounds present in foods—e.g., flavonoids found in fruits and vegetables, coffee, tea, chocolate, and wine—can alter CYP3A enzymes’ expression [15]. A prime example is the inhibition of CYP3A enzymes’ expression by components of grapefruit juice [12,16]. There is experimental evidence that retinoids can regulate the expression of CYP3A genes [17]. Certain diets, such as high-fat diets, can alter the expression of CYP3A genes [18], and it is likely that human dietary habits can affect basal expression of these genes [11]. 

Many of these substances are in turn metabolized by induced CYP3A enzymes, and this feedback mechanism implements detoxification of potentially harmful compounds [12]. 

### Members of the CYP3A Subfamily: Localization of Genes in the Genome and of Enzymes in Tissues of the Body

**Figure 1:**
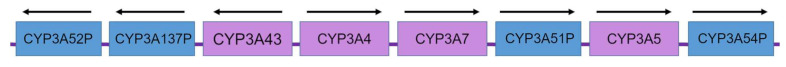
An outline of the human cytochrome P450 3A (*CYP3A*) locus on chromosome 7. Arrows indicate the orientation of open reading frames of genes. P: CYP3A pseudogenes [22].

To date, four functional enzymes belonging to the CYP3A subfamily have been identified in humans: CYP3A4, CYP3A5, CYP3A7, and CYP3A43 [5,6,8,9]. This subfamily is encoded by a 231 kbp cluster of four *CYP3A* genes in chromosomal region 7q21.1 (*CYP3A4*, *CYP3A5*, *CYP3A7*, and *CYP3A43*) and of several pseudogenes (Figure 1) [10,19,20,21]. Each gene contains 13 exons with conserved exon–intron boundaries [10,19]. The organization of the CYP3A locus indicates that it has arisen from duplications of an ancestral *CYP3A* cassette [12].

According to mass spectrometric analysis, proportions of proteins CYP3A4, CYP3A5, CYP3A7, and CYP3A43 in the total amount of CYP3A proteins are on average 85.4%, 5.4%, 3.4% and 5.8%, respectively [23]. CYP3A isoenzymes are expressed mainly in the liver and small intestine as well as in the kidneys, adrenal glands, lungs, brain, prostate, testes, placenta, pancreas, and skeletal muscles (Table 1) [5,6,8,9,11,14,20,24,25,26]. Expression of CYP3A enzymes in enterocytes is comparable to or may even exceed that in hepatocytes [12]. It is believed that CYP3A isoenzymes taken together constitute most of the protein amount of CYP enzymes in the liver and small intestine, and CYP3A4 accounts for the bulk of the CYP3A protein amount [12,23]. 

In the CYP3A subfamily, CYP3A4 is the major member participating in drug metabolism and is the predominant form of CYP3A in the liver (10–50%) and small intestine (40%) of adult humans. CYP3A4 shows the largest interindividual differences, by a factor of several tens to hundreds, in terms of mRNA and protein expression in the liver [23,27].

CYP3A4 in the liver is expressed more weakly in women than in men, as reported in ref. [28] or more strongly than in men, according to another report [29]. CYP3A4 is also expressed in the esophagus, duodenum, and colon (Table 1) [26]. Fetal CYP3A4 protein levels are extremely low in the first trimester but increase rapidly in the second and third trimesters of pregnancy [14]. *CYP3A4* mRNA expression shows 10-fold variation and increases with age after conception [30,31]. 

CYP3A5 is the most abundant and best studied among minor isoforms of CYP3A [6,8]. Unlike CYP3A4, functional CYP3A5 is expressed in approximately 70% of Africans and Afro-Americans and only in 20% of Eurasians [8,12,13]. Relatively large amounts of CYP3A5 are found in the intestines, kidneys, adrenal glands, prostate, and lungs [9,25]. CYP3A5 is the predominant CYP3A protein in human kidneys, lungs, blood, and pituitary and is also present in liver and intestinal tissues [5,26]. CYP3A5 also shows interindividual differences—from severalfold to hundreds of times—in terms of protein expression in the liver [23,32,33]. 

CYP3A5 expression is detectable in enterocytes in approximately 70% of adults [12]. CYP3A5 is expressed during the secretory phase in the endometrium, while CYP3A4 and CYP3A43 expression in this tissue is repressed by estrogen [28]. In the lungs, CYP3A5 is most abundant in bronchial and alveolar epithelia, bronchial glands, and alveolar macrophages [9]. CYP3A5 expression appears to vary throughout development [6]. 

CYP3A7 and CYP3A43 are underexpressed as compared to CYP3A4/5 [6,8]. Protein expression of CYP3A43 in human liver microsomes is ~15 times lower than that of CYP3A4 [23].

CYP3A7, a predominantly fetoplacental enzyme, is highly expressed in the liver and intestines of the embryo and fetus as well as in the endometrium and placenta, although it is detectable in the liver and small intestine of some adults [5,6,8,12,26,34]. Expression of CYP3A7 varies several-hundred-fold among individuals [30]. CYP3A7 is highly expressed during the first trimester of pregnancy and then its expression gradually declines [14]. 

Among the *CYP3A* genes, *CYP3A43* was the last to be identified. CYP3A43 expression is highest in the prostate (the organ with intensive metabolism of steroids) and in the brain: 170 times higher than CYP3A4 expression [35]. CYP3A43 is also found in the testes, liver, kidneys, placenta, and pancreas [5,6,8,9,14,20,24]. In the liver, *CYP3A43* mRNA levels vary up to 1000-fold among whites [14]. 

**Table 1:** Hepatic and extrahepatic expression profiles of human CYP3A4 and CYP3A5.

	CYP3A4		**CYP3A5**	
	Internal			
Liver	Abundance 68–155 pmol/mg RNA sequencing (RNA-seq): very high expression Microarray and RNA-seq: over-expressed	[23,36,37,38,39,40,41,42] [43] [44]	Abundance 2–5 (CYP3A5*3 allele) or 60–291 (CYP3A5*1 allele) pmol/mg Microarray and RNA-seq: overex-pressed RNA-seq: high expression	[45] [44] [43]
Small intestine	RNA detected by real-time PCR; protein detected; enzyme activity detected	[46,47,48]	RNA detected by real-time PCR; protein detected; enzyme activity detected	[41,47,48]
	Microarray and RNA-seq: overexpressed	[44]	Microarray and RNA-seq: overexpressed	[44]
	RNA-seq: very high expression	[43]	RNA-seq: high expression	[43]
Duodenum	RNA-seq: very high expression	[43]	RNA-seq: high expression	[43]
Colon	Microarray and RNA-seq: detected RNA-seq: low expression	[44] [43]	Microarray and RNA-seq: detected RNA-seq: high expression	[44] [43]
Esophagus	RNA-seq detected RNA-seq: low expression	[44] [43]	RNA-seq detected RNA-seq: moderate expression	[44] [43]
Stomach	RNA-seq detected RNA-seq: low expression	[44] [43]	RNA-seq detected RNA-seq: high expression	[44] [43]
Gall bladder	RNA-seq: low expression	[43]	RNA-seq: high expression	[43]
Kidney	RNA detected by real-time PCR; protein detected; enzyme activity detected	[43,44,46,49]	RNA detected by real-time PCR; protein detected; enzyme activity detected	[46,49]
	Microarray and RNA-seq: detected	[44]	Microarray and RNA-seq: detected	[44]
	RNA-seq: low expression	[43]	RNA-seq: moderate expression	[43]
Lung	RNA detected by real-time PCR	[46]	RNA detected by real-time PCR	[46]
	Microarray and RNA-seq: detected	[44]	Microarray and RNA-seq: detected	[44]
	RNA-seq: low expression	[43]	RNA-seq: moderate expression	[43]
Adipocyte, adipose tissue	Microarray and RNA-seq: detected RNA-seq: low expression	[44] [43]	Microarray and RNA-seq: detected RNA-seq: low expression	[44] [43]
Spleen	RNA-seq detected RNA-seq: extremely low expression	[44] [43]	RNA-seq detected RNA-seq: low expression	[44] [43]
Bladder	RNA-seq: low expression	[43]	RNA-seq: moderate expression	[43]
Secretory				
Pancreas	RNA-seq detected RNA-seq: low expression	[44] [43]	RNA-seq: moderate expression	[43]
Adrenal gland	RNA detected by real-time PCR	[46]	RNA detected by real-time PCR	[46]
	Microarray and RNA-seq: detected	[44]	Microarray and RNA-seq: detected	[44]
	RNA-seq: moderate expression	[43]	RNA-seq: moderate expression	[43]
Pituitary	RNA-seq detected	[44]	RNA-seq detected	[44]
Thyroid gland	Microarray and RNA-seq: detected RNA-seq: extremely low expression	[44] [43]	Microarray and RNA-seq: detected RNA-seq: low expression	[44] [43]
Salivary gland	Microarray and RNA-seq: detected RNA-seq: extremely low expression	[44] [43]	Microarray and RNA-seq: detected RNA-seq: low expression	[44] [43]
Breast	RNA-seq detected	[44]	RNA-seq detected	[44]
Skin/keratinocytes	RNA detected by real-time PCR Microarray and RNA-seq: detected RNA-seq: low expression	[50] [44] [43]	Microarray and RNA-seq: detected RNA-seq: high expression	[44] [43]
Nervous				
Brain (cortex)	Not detectable	[51]	RNA detected by real-time PCR; protein detected	[52,53]
	RNA-seq detected	[44]	Microarray and RNA-seq: detected	[44]
	RNA-seq: extremely low expression	[43]	RNA-seq: low expression	[43]
Cerebellum	Microarray and RNA-seq: detected	[44]	Microarray and RNA-seq: detected	[44]
Retina	Microarray: detected	[44]	Microarray: detected	[44]
Spinal cord	Microarray and RNA-seq: detected	[44]	Microarray and RNA-seq: detected	[44]
Tibial nerve	RNA-seq detected	[44]	RNA-seq detected	[44]
Muscle				
Heart	Not detectable	[54]	Not detectable	[54]
	Microarray and RNA-seq: detected	[44]	Microarray and RNA-seq: detected	[44]
	RNA-seq: extremely low expression	[43]	RNA-seq: low expression	[43]
Artery	RNA-seq detected	[44]	RNA-seq detected	[44]
Smooth muscle	Microarray: detected	[44]	Microarray: detected	[44]
Skeletal muscle	Microarray and RNA-seq: detected	[44]	Microarray and RNA-seq: detected	[44]
Reproductive				
Ovary	Not quantifiable	[43]	RNA detected by real-time PCR	[46]
	Microarray and RNA-seq: detected	[44]	Microarray and RNA-seq: detected	[44]
	RNA-seq: low expression	[43]	RNA-seq: low expression	[43]
Uterus	Microarray and RNA-seq: detected	[44]	Microarray and RNA-seq: detected	[44]
Endometrium	RNA-seq: low expression	[43]	RNA-seq: moderate expression	[43]
Placenta	Not detectable	[46]	RNA detected by real-time PCR	[46]
	Microarray and RNA-seq: detected	[44]	Microarray: detected	[44]
	RNA-seq: not detectable	[43]	RNA-seq: low expression	[43]
Prostate	RNA detected by real-time PCR	[46]	RNA detected by real-time PCR	[46]
	Microarray and RNA-seq: detected	[44]	Microarray and RNA-seq: detected	[44]
	RNA-seq: low expression	[43]	RNA-seq: moderate expression	[43]
Testis	RNA detected by real-time PCR	[46]	RNA detected by real-time PCR	[46]
	Microarray and RNA-seq: detected	[44]	Microarray and RNA-seq: detected	[44]
	RNA-seq: low expression	[43]	RNA-seq: low expression	[43]
Immune				
Lymph node	Microarray: detected RNA-seq: low expression	[44] [43]	Microarray and RNA-seq: detected RNA-seq: low expression	[44] [43]
Bone marrow	Microarray: detected RNA-seq: extremely low expression	[44] [43]	Microarray: detected RNA-seq: low expression	[44] [43]
Whole blood	Microarray and RNA-seq: detected	[44]	Microarray and RNA-seq: detected	[44]
White blood cells	Not detected	[44]	RNA-seq: detected	[44]
Thymus	Microarray: detected	[44]	Microarray: detected	[44]
Appendix	RNA-seq: low expression	[43]	RNA-seq: moderate expression	[43]

## 2. Mechanisms of CYP3A Regulation

### 2.1. Constitutive Regulation of CYP3A4 Transcription

**Figure 2:**
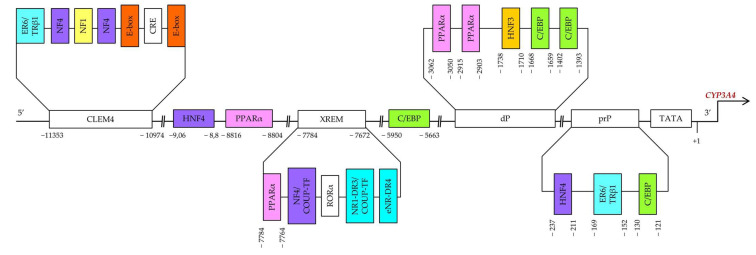
Binding sites in the upstream part of *CYP3A4*. prP: proximal promoter, dP: distal promoter, XREM: a distal element called xenobiotic-responsive enhancer module, CLEM4: a distal element called constitutive liver enhancer module 4.

Constitutive regulation of *CYP3A4* transcription, both positive and negative, is mediated by hepatocyte nuclear factor 4α (HNF4α) and other hepatic transcription factors, including HNF1α and HNF3γ [55,56,57,58,59], CCAAT/enhancer-binding proteins alpha and beta (C/EBPα and C/EBPβ), and upstream transcription factor 1 (USF1) [10,60] via binding to three major *cis*-acting modules: constitutive liver enhancer module 4 (CLEM4) (positions −11.4 to −10.5 kbp), the distal xenobiotic-responsive enhancer module (XREM) (−7.2 to −7.8 kbp) and the proximal promoter (prP) (Figure 2) [10].

When bound to DNA, HNF4α attracts transcription coactivators and other accessory proteins and positively regulates the expression of target genes. In the liver, HNF4α is located exclusively in the nucleus and regulates the constitutive expression of a large number of target genes, including *CYP3A4* [61].

HNF4α is required for the active epigenetic state of the enhancers that have been shown to increase gene transcription in mouse hepatocytes. In hepatocytes, HNF4α binds almost exclusively to active enhancers marked by histone modifications [lysine 4 histone 3 monomethylation (H3K4me1) and histone 3 lysine 27 acetylation (H3K27ac)] and DNA hydroxymethylation (5hmC), indicating a major role for HNF4α in transcription activation. Mice lacking HNF4α protein in hepatocytes show a decrease in the amounts of both H3K27ac and 5hmC in HNF4α-associated DNA regions. In terms of the mechanism, when bound to HNF4α, 5hmC requires an interaction of this protein with a ten-eleven translocation protein 3 (TET3) responsible for the oxidation of 5mC to 5hmC. Moreover, HNF4α regulates TET3 expression in the liver by direct binding to an enhancer region [62].

Functional binding sites for HNF4α [direct repeat 1 (DR1)] are located in an XREM spanning positions −7783 to −7771 bp [57], in the far distal region at −9.06/−8.8 kbp, and in the proximal promoter region at the −237/−211 bp site [58]. HNF4α determines pregnane X receptor (PXR)-mediated and constitutive androstane receptor (CAR)-mediated induction of *CYP3A4* by xenobiotics. Disruption of the HNF4α-binding site in XREM has been found to reduce basal and inducible CYP3A4 expression in mice [57].

A more recent study on primary human hepatocytes and on the HepG2 cell line revealed that selective disruption of the DR1 element in XREM causes only minor changes in the level of *CYP3A4* transactivation [63]. Furthermore, the relevance of this HNF4α-binding site has been further questioned by research article indicating that the XREM region does not promote HNF4α activation in the context of the natural 5′ regulatory upstream region of *CYP3A4*.

It has been shown that an interaction of two regions (at −9.06/−8.8 kbp and −237/−211 bp) is required for maximal expression activation by HNF4α. The effect of HNF4α is counteracted by chicken ovalbumin upstream promoter transcription factor (COUP-TF) II upstream of the promoter; this protein binds to one of the DR1 motifs. Furthermore, the activation of *CYP3A4* via the DR1 element in the proximal promoter is dependent on an additional factor that binds near position −189 bp. Physiological significance of this position for HNF4α activation in vivo is supported by the presence of binding activity in the small intestine similar to that in LS174T cells. These results support the hypothesis that HNF4α directly regulates basal *CYP3A4* expression, at least in the gut [58].

Hepatocyte nuclear factor 4α antisense RNA 1 (HNF4A-AS1), which is a long noncoding RNA (lncRNA), is reported to be a negative regulator of basal and rifampicin-induced expression of nuclear receptors and downstream P450 enzymes. In Huh7 cells, a knockdown of HNF4A-AS1 results in increased expression of HNF4α, PXR, and P450 enzymes (including CYP3A4) both at baseline and in the context of rifampicin-induced expression. By contrast, overexpression of HNF4A-AS1 decreases baseline expression of CAR, aryl hydrocarbon receptor, PXR, and some P450 enzymes. Of note, substantially attenuated induction of *PXR*, *CYP1A2*, *CYP2C8*, *CYP2C19*, and *CYP3A4* by rifampicin was also observed in Huh7 cells transfected with an HNF4A-AS1–expressing plasmid. In addition, negative feedback of HNF4α on HNF4A-AS1–mediated gene expression was confirmed by a loss-of-function experiment. The *CYP3A4* promoter enhances the expression of CYP3A4 after the HNF4A-AS1 knockdown. In general, histone modifications promote downregulation of nuclear receptors and some P450 enzymes by HNF4A-AS1 at basal and drug-induced levels [64].

The region spanning positions from −11.4 to −10.5 kbp (CLEM4) plays an important part in constitutive activation of the *CYP3A4* gene. HNF1α, HNF4α, USF1, and activating protein-1 (AP-1) have been demonstrated to interact with CLEM4. Additionally, introduction of mutations into their binding sites showed that almost all these sites are required for maximal enhancer activity [65]. HNF3 may facilitate the association of other transcription factors with their binding sites through chromatin remodeling [66].

C/EBPs are key transcription factors involved in constitutive expression of several cytochrome P450 genes in the liver [67]. Genes *C/EBPα* and *C/EBPβ* can produce several N-terminally truncated isoforms of the protein as a result of post-transcriptional mechanisms [68]. CYP3A4 is modulated by the following factors: C/EBPα, a C/EBPβ isoform called liver-enriched transcriptional activator protein (LAP, ~35 kDa), and a C/EBPβ isoform called liver-enriched transcriptional inhibitory protein (LIP, ~20 kDa) [69,70]. 

LAP is a transcriptional activator in many genetic systems, whereas LIP is considered a functional antagonist of LAP. Because the low-molecular-weight LIP isoform lacks most of the transactivation domain but contains DNA-binding domains and dimerization domains, it has been suggested that it acts as a dominant-negative regulator of the full-length C/EBP protein and of LAP [71,72,73]. The LAP/LIP ratio controls constitutive and inducible expression of *CYP3A4* and may contribute to various CYP3A4 phenotypes in the human population [60].

It has been demonstrated in HepG2 cells that functional C/EBP-binding sites are present in the proximal promoter of *CYP3A4* at positions −121/−130 and in the distal promoter of *CYP3A4* at positions −1393/−1402 and −1659/−1668 [59]. There is also a C/EBP-binding distal enhancer site between positions −5950 and −5663 bp in the 5′ flanking region of *CYP3A4*. A strong competitive effect between LAP and LIP has been found on a distal *CYP3A4* sequence. C/EBP–LAP and LIP interact with a 288 bp distal enhancer site in the region near −5.95 kbp and modulate *CYP3A4* expression in hepatic and nonhepatic cells [60].

### 2.2. Regulation of CYP3A4 Transcription

CYP3A4 expression is modulated by various mechanisms involving nuclear receptors, hormones, xenobiotics, and signaling molecules. *CYP3A4* is regulated by a large number of xenobiotics, including many drugs, endogenous compounds, and many hormones, such as triiodothyronine, dexamethasone, and growth hormone [74].

**Figure 3:**
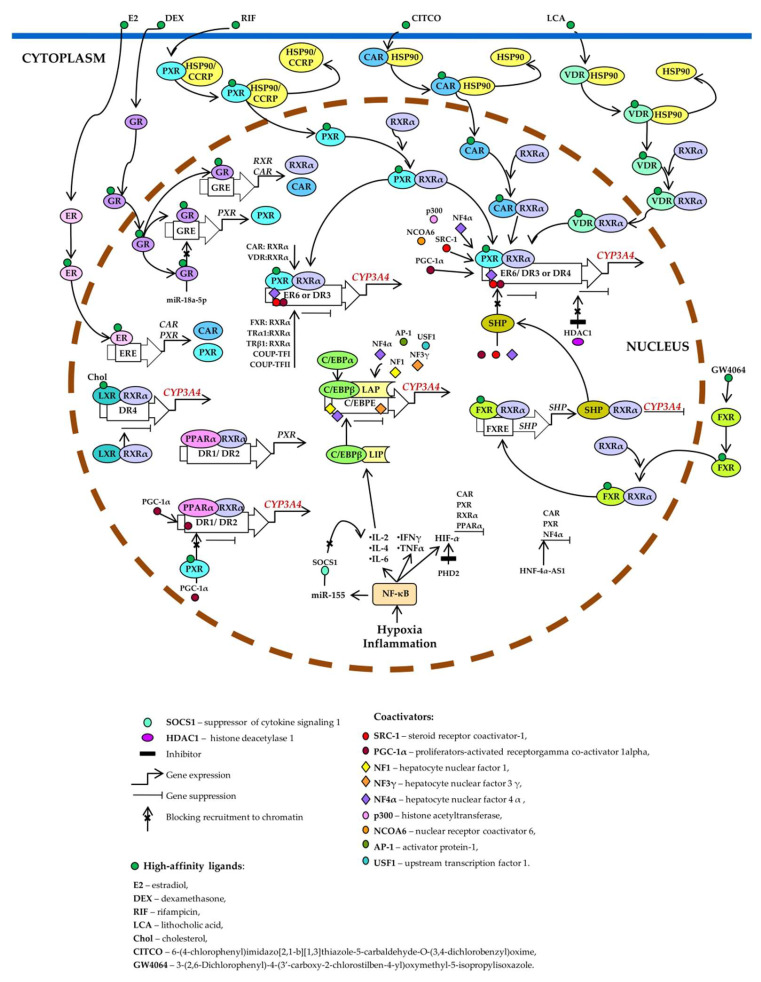
Transcriptional regulation of CYP3A4. Pregnane X receptor (PXR), constitutive androstane receptor (CAR), and vitamin D receptor (VDR) control basal and inducible expression of CYP3A4 through competitive binding to the same set of response elements (everted *repeats* 6, ER6; direct repeats DR3, and DR4). PXR, CAR, or VDR unbound by a ligand is located in the cytoplasm as a complex with heat shock protein 90 (HSP90) or cytoplasmic constitutive active/androstane receptor retention protein (CCRP). When activated by a ligand, each of them forms a heterodimer with retinoid X receptor α (RXRα), relocates to the nucleus, binds to a response element, recruits coactivators, and activates *CYP3A4* transcription. Estrogen receptor (ER) and glucocorticoid receptor (GR) raise CYP3A4 expression by enhancing the expression of CAR, RXRα, and PXR. Ligand-activated farnesoid X receptor (FXR) upregulates small heterodimer partner (SHP), which prevents the recruitment of coactivators to chromatin and/or forms heterodimers with RXRα, thereby inhibiting CYP3A4 expression. Histone deacetylase 1 (HDAC1) inhibition by carbamazepine downregulates CYP3A4. Liver X receptor (LXR) forms a heterodimer with RXRα that then binds to DR4 in the target gene, thus repressing its expression. After the binding of a ligand to LXR or RXR, the heterodimer changes its conformation, which leads to a release of corepressors and the recruitment of coactivators. This event causes transcription of a target gene (peroxisome proliferator-activated receptor alpha; *PPARα*), its protein product binds as a PPARα–RXRα heterodimer to motifs DR1 and DR2 and enhances the transcription of *CYP3A4* and *PXR*. Ligand-activated PXR suppresses PPARα-dependent gene expression by inhibiting peroxisome proliferator-activated receptor-gamma coactivator 1 alpha (PGC-1α) recruitment. Hypoxia and inflammation induce the activity of nuclear factor kappa B (NF-κB) and promote a release of the cytokines that increase the transcription of CCAAT enhancer-binding protein beta (*C/EBPβ*) and the translation of *C/EBPβ-LIP* mRNA. C/EBPβ-LIP competes with C/EBPα and C/EBPβ-LAP for binding to response elements in the promoter of *CYP3A4*, thus lowering its expression. NF-κB activates miR-155, which directly targets mRNAs of suppressors of cytokine signaling proteins (especially suppressor of cytokine signaling 1: SOCS1) thereby inhibiting obligatory negative feedback regulation of inflammatory responses. *Abbreviations*. C/EBPβ-LAP: a C/EBPβ isoform called liver-enriched activator protein; C/EBPβ-LIP: a C/EBPβ isoform called liver-enriched inhibitory protein; COUP-TFI: chicken ovalbumin upstream promoter transcription factor I; COUP-TFII: chicken ovalbumin upstream promoter transcription factor II; DR1, DR2, DR3, DR4, and ER6: AG(G/T)TCA-like direct repeats separated by 1, 2, 3, or 4 bases, respectively, and an inverted repeat separated by 6 bases; ERE: ER-responsive element; FXRE: FXR-responsive element, GRE: GR-responsive element; HIF-1α: hypoxia-inducible factor 1-α; HNF-4α-AS1: hepatocyte nuclear factor 4α-antisense-RNA 1; IL-2, -4, or -6: interleukin 2, 4 or 6; INFγ: interferon γ; PHD2: prolyl hydroxylase domain-containing protein 2; TNF: tumor necrosis factor; TRα1: thyroid hormone receptor-α1; TRβ1: thyroid hormone receptor-β1.

Xenobiotic- and endobiotic-mediated CYP3A4 induction is indirect and entails activation of such ligand-dependent nuclear receptors as PXR, CAR, VDR, glucocorticoid receptor (GR) α, estrogen receptor (ER) α, bile acid receptor (farnesoid X receptor; FXR), oxysterol receptor (liver X receptor; LXR), and peroxisome proliferator-activated receptor alpha (PPARα) [10,11,14,27,75,76,77,78,79,80,81,82,83,84,85,86,87,88,89] as well as by binding to the three major *cis*-acting modules: CLEM4, distal XREM, and prPXRE (Figure 3) [10]. 

Nuclear receptors participating in the regulation of *CYP3A4* share protein partners, ligands, DNA-sensing elements, and target genes, thereby forming a complex regulatory network by which the cell adapts to changes in its chemical environment [9,14].

PXR is considered the most important and critical factor that determines the activity and expression of the hepatic enzyme CYP3A4 [90,91]. PXR binds to the ligand and is then translocated into the nucleus. There, it heterodimerizes with the retinoid X receptor (RXR) and enhances CYP3A transcription by binding to AG(G/T) TCA-like direct repeats separated by 3 or 4 bases (DR3 and DR4, respectively) and to everted repeats separated by 6 bases (ER6) [90,91,92]. Heterodimer PXR–RXRα binds to ER6 in the proximal promoter [93], to DR3 in XREM [76], to ER6 in a far distal enhancer module [91], and to a DR4 motif [94,95].

RXR plays a central role in the regulation of many endobiotic and xenobiotic metabolic pathways and represents an additional node of control over PXR-mediated regulation of CYP3A4 (especially in RXR-deficient tissues [6]) by forming a heterodimer with numerous nuclear receptors for the modulation of enzymes.

CAR is a nuclear receptor known primarily to mediate CYP2B induction. PXR and CAR can be activated by the same sets of compounds such as phenobarbital and TCPOBOP [96,97,98]. Although PXR binds strongly to the DR4, DR3, or ER6 motif in the CYP3A4 promoter [99,100], CAR binds only weakly to the proximal ER6 motif in CYP3A4 [100,101], resulting in a preference of CAR for CYP2B6 over CYP3A4 in humans.

VDR is a member of the nuclear receptor superfamily of transcription regulators and mediates various biological effects of 1,25-dihydroxyvitamin D3 by modulating the transcription of target genes [102].

PXR, CAR, and VDR use the same set of binding sites in the 5′ regulatory region of CYP3A4 gene [12,14] and control CYP3A4 expression through competitive interaction with the same response elements in DNA (ER6, DR3, and DR4) [103]. In the absence of a ligand, CAR, DVR and PXR are located in the cytosol in complex with chaperones. After ligand binding, the PXR or CAR are released from the complex and translocated to the nucleus, where they form a heterodimer with RXRα. VDR is located in the cytosol of vitamin D–sensitive cells, and upon binding to 1,25(OH)_2_D_3_, the complex forms the VDR–RXR heterodimer, which relocates to the cell nucleus [12,14].

Aside from PXR, CAR, and VDR, RXRα forms heterodimers with other nuclear receptors that affect the expression and induction of *CYP3A4*. VDR, FXR, LXRα, CAR, and thyroid hormone receptors TRα1 and TRβ1 bind to PXR-binding sites ER6 in the proximal region and to DR3 in XREM. COUP-TFI, which is a nuclear receptor of steroid hormone receptors, binds as a homodimer to both PXR-specific motifs but preferentially binds to distal DR3. On the contrary, COUP-TFII is able to bind only as a homodimer to the distal element DR3. TRβ1 binds as a homodimer exclusively to element ER6 [104]. 

FXR is a primary bile acid receptor expressed in hepatic and intestinal tissues, where it regulates the uptake, metabolism, and removal of bile acids. In the absence of a ligand, FXR is in complex with a corepressor. After activation by the ligand, FXR forms a heterodimer with RXR and binds to the DNA sequence (FXR response element—FXRE) in the promoter of target genes and activates transcription [105]. 

There are two types of FXR’s participation in *CYP3A4* expression. Bile acid–activated FXR induces CYP3A4 expression and phase I hydroxylation reactions (xenobiotic metabolism) [84,106,107]. On the other hand, FXR activation represses *CYP3A4* expression by inducing FXR target genes that repress the activation of transcription: SHP (small heterodimeric partner) in the liver [108] and fibroblast growth factor in the small intestine [109].

LXRα and LXRβ (liver X-receptors α and β) form heterodimers with RXRα to function as transcriptional regulators [110]. Heterodimers LXR–RXRα bind to an LXR-responsive DNA element consisting of a direct repeat of the core AGGTCA sequence separated by four nucleotides (DR4), thereby enhancing target gene expression [111].The specific involvement of LRX in the activation or suppression of gene transcription depends on the type of cells and genes. In the absence of a ligand, the LXR heterodimer with RXR inhibits transcription due to the N-CoR corepressor (nuclear receptor corepressor) and SMRT (silencing mediator of retinoic acid and thyroid hormone receptor) [110,111,112,113]. After ligand activation and dissociation with corepressors, coactivators are recruited and transcription is stimulated [114].

PPARα is an important transcription factor regulating genes encoding endo- and xenobiotic-metabolizing and lipid-metabolizing enzymes. As heterodimer PPARα–RXRα, PPARα binds to motifs DR1 and DR2 [direct repeats of the AG(G/T)TCA half-site separated by 1 and 2 nucleotides, respectively]. PPARα–RXRα specifically binds to three DR1-like motifs and one DR1/DR2 motif (PBR-I, -II, and -III) at positions −2915/−2903 and −3062/−3050 as well as −7784/−7764 and −8816/−8804 [79].

PPARα activates *PXR* expression by binding to the region −1514 to −1321 bp. Moreover, forced overexpression of ERα or GRα exerts a positive effect on the activity of a *PXR* reporter construct carrying a 2.2 kbp proximal promoter sequence [115,116,117].

Experimental data point to redundancy as well as cooperativity of at least three functional PPARα-dependent sites in an *CYP3A4* enhancer that mediate both constitutive and inducible transactivation. Presumably, the binding regions upstream of CYP3A4 are permanently occupied by PPARα, and a cofactor environment determines whether the repression or activation of *CYP3A4* transcription takes place after ligand binding. Therefore, the addition of a chemical ligand does not lead to an enhancement of the binding but rather to a greater release of corepressors and thus an increase in transcription [79].

Less is known about the regulation of the *CYP3A5* gene, although it is likely that genes *CYP3A4* and *CYP3A5* share a common HNF4α-dependent pathway regulating constitutive expression [8,9,11,78]. Unlike *CYP3A4*, the *CYP3A5* gene sequence does not contain a distal region of XREM (from position −7836 to −7208 bp), and this may be the reason for the difference in expression regulation between *CYP3A4* and *CYP3A5* [14].

It has been shown that CAR and PXR activate the *CYP3A5* promoter in hepatocytes and intestines of carriers of allele CYP3A5*1 [118]. According to other data, basal expression of *CYP3A5* in extrahepatic tissues (intestines, kidneys, lungs, and prostate) does not depend on PXR [11,118]. CYP3A5 is the main enzyme of the CYP3A subfamily in the lungs, and its induction in this organ, unlike in the liver, is triggered only by glucocorticoids (not by typical CYP3A inducers) [6,12]. In lung adenocarcinoma A549 cells, CYP3A5 is activated by glucocorticoids via a GR [9]. 

Expression of CYP3A5 in organs devoid of PXR appears to be mediated by the loss of a suppressive YY1-binding element in the *CYP3A5* promoter [25]. YY1 is a ubiquitous transcription factor belonging to a class of proteins having clusters of GLI-Kruppel zinc finger domains taking part in the modulation of transcription of many genes; YY1 can function either as a transcriptional activator or as a repressor, as seems to be the case for *CYP3A* genes [25,119]. The loss of YY1-mediated transcriptional repression may contribute to the expansion of tissue specificity of *CYP3A5* in the absence of induction. For example, the expression of CYP3A5 in the kidneys promotes salt and water retention mediated by cortisol metabolite 6β-hydroxycortisol, which arises under the action of CYP3A5; this effect can be useful in hot climates. This phenomenon may explain the high prevalence of polymorphism-dependent *CYP3A5* expression in the kidneys of most Africans [25,120].

Nuclear receptors CAR and PXR play a key role in the induction of *CYP3A7* [121]. When the proximal ER6 motif of *CYP3A7* was compared with this motif of CYP3A4, two nucleotide mutations (−169G → −168T and −161A → −160T) were found, which may account for the reduced ability of complexes PXR–RXRα and VDR–RXRα to recognize and bind the *CYP3A7* promoter and to activate this gene’s transcription [122]. In addition, compared to CYP3A4 and CYP3A5, the ligand-binding affinity and catalytic ability of CYP3A7 are significantly weaker due to reduced structural plasticity of the CYP3A7 protein [34]. 

There are virtually no data on the mechanisms of regulation of *CYP3A43*. Four *cis*-regulatory elements are known that control *CYP3A4* transcription and interact with the *CYP3A4* promoter, one of which (R2) overlaps with known enhancers CLEM4 or XREM, and deletion of R4 increases *CYP3A4* expression while decreasing *CYP3A43* expression. The single-nucleotide polymorphism (SNP) rs62471956 in the R4 region correlates with higher expression of *CYP3A43* and lower expression of *CYP3A4* [123].

Thus, most CYP3A inducers act through transcriptional activation [9,11,14]. CYP3A isoforms and nuclear receptors involved in their regulation are subject (as part of post-transcriptional regulation) to ubiquitination (CYP3A4 and CYP3A5) and phosphorylation (CYP3A4 and PXR) [11], whereas post-translational regulation of CYP3A enzymes consists of the stabilization of CYP3A mRNAs and proteins [6,11]. 

Molecular mechanisms of induction may differ among the four major human *CYP3A* genes and among their polymorphic variants owing to differences in their structure, and the mechanisms can also differ among different tissues, possibly because of different ratios of crucial protein factors. This complexity is a consequence of the wide range of CYP3A ligands and of nuclear receptors mediating the induction of *CYP3A* genes [9,14].

### 2.3. Negative Regulation of CYP3A Enzymes

Suppression of *CYP3A* genes is mediated by the activation of nuclear factor kappa B (NF-κB) [124]. In addition, tumor necrosis factor (TNF) attenuates PXR-mediated transcriptional activation induced by rifampicin in vitro [124,125]. Proteins p53, NF-κB, and C/EBP-LIP are involved in the repression of *CYP3A4* gene activity [9]. NF-κB activation plays an important role in the suppression of *CYP3A* genes by disrupting the binding of the PXR–RXRα complex to DNA [126]. Cytokine-mediated downregulation of *CYP3A4* during the inflammatory response via the Janus kinase (JAK)–signal transducer and activator of transcription (STAT) pathway is of great importance [10].

Major cytokines—interleukin (IL)-1β, IL-1α, IL-6, and TNF—downregulate genes *CYP3A4* and *CYP3A5* in human hepatocytes and *Cyp3a* genes in murine and rat hepatocytes [127,128,129]. The level of *CYP3A4* mRNA is significantly reduced by IL-1β, IL-6, and TNF in a 3D culture of human hepatocarcinoma FLC-4 cells [128,130]. 

TNF attenuates PXR-mediated transcriptional activation induced by rifampicin. Induction of CYP3A4-luciferase activity of a cotransfected CYP3A4-luciferase construct and of a PXR expression plasmid by rifampicin is significantly weakened by TNF [131]. IL-6 appears to be a key factor for downregulation of the activity of CYP3A enzymes during inflammation [130,132,133]. It has been shown that IL-6 negatively regulates CYP3A4 with the participation of glycoprotein receptor gp130 and of the C/EBPβ signaling pathway [127].

Endoplasmic-reticulum stress is reported to decrease PXR expression and *CYP3A4* induction in primary human hepatocytes and human HepG2 cells. Apparently, the reason is the suppression of HNF-4α and LAP during endoplasmic-reticulum stress, whereas overexpression of HNF-4α or LAP restores PXR expression. An analyzed sequence—located in the *PXR* promoter region 2 adjacent elements (recognized by HNF-4α and C/EBPs)—is also responsive to IL-6 (as detected via luciferase activity), indicating a functional relation between endoplasmic-reticulum stress and proinflammatory cytokine signaling [134].

Another mechanism of *CYP* genes’ suppression involving the serotonergic system has been described in a rat model of diethylnitrosamine-induced liver failure [135]. Namely, serotonergic-system dysfunction caused by a tryptophan-free diet lowered the levels of proinflammatory cytokines TGF-β and IL-1β but increased the amount of anti-inflammatory cytokine IL-4. Triggering of the repressive mechanism IL-4–JAK1–STAT6– suppressor of cytokine signaling 1 (SOCS1) in the JAK2–STAT5b-mediated signal transduction pathway and proline-rich, extensin-like receptor kinase (pERK)1/2–GR–STAT6 signal transduction pathway led to the repression of *CYP2C11* and of *CYP3A* genes [135].

### 2.4. An Additional Level of Regulation of CYP3A Genes 

#### 2.4.1. Epigenetic Mechanisms of CYP3A Gene Regulation

Expression of CYP genes is influenced by epigenetic factors including histone modifications, DNA methylation, and regulation by noncoding RNAs [136]. 

Histone proteins undergo post-translational modifications such as acetylation, methylation, phosphorylation, ubiquitination, and some others, which can affect gene expression by changing chromatin architecture and DNA accessibility for transcription [136]. For instance, histone modifications may participate in the *CYP3A4* and *CYP3A7* expression changes that occur in the embryonic period and in the first 2 years after birth. This mechanism seems to be important for CYP3A4, although other mechanisms may play a greater role in the regulation of *CYP3A7* during ontogenesis [137].

DNA methylation at CpG sites in a promoter region results in transcriptional repression and has numerous effects on the expression of CYP genes in various organs [136]. For example, a DNA methylation inhibitor called 5-aza-2-deoxycytidine increases the expression of *PXR* and *CYP3A4* in HepG2 cells in a concentration- and time-dependent manner. Conversely, methylation of a *CYP3A4* enhancer inhibits the PXR-mediated transcriptional activity of the *CYP3A4* gene and the binding of PXR to the *CYP3A4* promoter. As a result of methylation of the *CYP3A4* promoter and enhancer, rifampicin stops enhancing *CYP3A4* expression [138].

Noncoding RNAs—miRNAs and lncRNAs—have been identified as epigenetic modulators of expression of CYP genes [136]. Non-coding microRNAs can mediate transcriptional and post-transcriptional regulation of CYP3A in two ways. Firstly, by directly acting on the 3′-untranslated region (3′UTR) of the CYP3A4 mRNA or, secondly, by binding to the 3′UTR mRNA of nuclear receptor genes involved in the regulation of the CYP gene, thus acting on CYP3A4 activators or repressors [139,140].

For example, it has been shown that in non-alcoholic fatty liver disease, changes in the levels of miR-150-5p and miR-200a-3p [141] can post-transcriptionally suppress CYP3A4 [138,140,141,142], whereas miR-34a, miR-30c-1-3p and miR-27b suppress CYP3A4, affecting RXRa, PXR and VDR [136,140]. In a small clinical study, miR-27b expression was found to correlate with blood plasma levels of 4β-hydroxycholesterol, thus revealing an association between *CYP3A* and miR-27b in vivo [142]. Furthermore, in a study on 105 male patients treated with alprazolam, a significant association was found between the urinary metabolic ratio of 6β-hydroxycortisol to cortisol and miR-27b in plasma [143]. Indirect regulation of *CYP3A4* by miR-27b may be due to miR-27b binding to *VDR* mRNA, resulting in a decrease in CYP3A4 protein expression [140].

MiR-148a has been found to influence inducible and constitutive expression of CYP3A4 by downregulating PXR [144]. From data on the impact of miRNAs on CYP3A5 expression, it is known that miR-543 directly binds to the 3′UTR of *CYP3A5* mRNA and reduces the expression of the CYP3A5 protein [145]. Recently, in a study on the impact of basal CYP3A5 expression in donor liver transplants on the metabolism of an immunosuppressant (tacrolimus), it was demonstrated that miR-26b-5p directly regulates CYP3A5 expression, whereas miR-29a-5p, miR-99a-5p, and miR-532-5p target regulatory molecules HNF4α, NR1I3, and NR1I2, respectively [146].

There is research suggesting that lncRNAs may also contribute to the regulation of drug metabolism by acting on miRNAs [147]. The most common mechanism of CYP gene regulation mediated by lncRNAs is based on interactions between a lncRNA and mRNA of regulatory proteins. LncRNAs expressed near genomic loci of nuclear-receptor genes can modulate CYP gene expression along with expression of the corresponding nuclear receptors. Genes of antisense lncRNAs HNF1A-AS1 and HNF4A-AS1 are located adjacent to the genes that regulate CYP gene expression, i.e., genes of transcription factors HNF1α and HNF4α, respectively, thereby acting as coregulators of transcription of their target genes, including *CYP3A4* [136,148]. 

#### 2.4.2. Genetic Polymorphisms of CYP3A Genes and Their Influence on the Activity of CYP3A Enzymes 

Although members of the CYP3A subfamily share high identity of amino acid and DNA sequences, their tissue- and age-specific expression patterns and substrate specificity differ considerably [137]. Genetic polymorphisms are a major cause of inter-individual differences in CYP3A expression and function [3,149]. 

Most *CYP3A4* polymorphic variants are SNPs [26]. To date, 9815 SNPs have been found in the human *CYP3A* gene as documented in the National Center for Biotechnology Information (NCBI) SNP database (http://www.ncbi.nlm.nih.gov/snp). The following is known about the functional effects of SNPs of *CYP3A* genes.

*CYP3A4*, which codes for CYP3A4, the main enzyme of the CYP3A subfamily, has several SNPs correlating with metabolic activity of CYP3A4 [150,151], for example, allele CYP3A4*1B (rs2740574, −392A>G) located in the promoter [152] and allele CYP3A4*1G (rs2242480, 20230C>T) both increasing the activity of the CYP3A4 enzyme [153,154,155] and allele CYP3A4*22 (rs35599367, 15389 C>T) situated in intron 6 and reducing CYP3A4 enzymatic activity [153,155,156]. SNP CYP3A4*22 is known to double the production of a nonfunctional CYP3A4 variant [26,149,157,158]. Most other CYP3A4 polymorphisms have very low frequencies and their phenotypic effects are weak and often inconsistent [12,26,149]. On the other hand, CYP3A4*6, *17, *20, and *26 have been shown to correlate with reduced enzymatic activity due to a loss of function [159,160,161,162].

Another common allele that affects drug metabolism and hormone metabolism is CYP3A5*3 (rs776746) [157]. Hepatic CYP3A5 protein levels are low or undetectable in most whites. The reason is a common A>G mutation in intron 3 of the *CYP3A5* gene (allele CYP3A5*3) [12]. The low CYP3A5 protein expression in CYP3A5*3 carriers is attributed to defective mRNA splicing and reduced synthesis of the functional protein [163]. Individuals with at least one CYP3A5*1 allele are known to express CYP3A5, whereas individuals with the CYP3A5*3/*3 genotype are known to not express CYP3A5 [164].

CYP3A7 is expressed in the liver at a much lower level in 90% of adults than in the fetus, but a high-expression phenotype is seen in 10% of adults. Two-thirds of the latter carry a promoter allele called CYP3A7*1C (*rs45446698*, located in the promoter) or (less commonly) CYP3A7*1B. Allele CYP3A7*1C induces overexpression of the fetal *CYP3A7* gene in adults, influences sex hormone levels [165] and is an exclusive marker of high CYP3A7 expression in the gut [26]. 

For *CYP3A43*, genetic variant rs472660 in the intron is known to be associated with a response to antipsychotic drug olanzapine, and this gene’s functional SNP marker rs680055 correlates with this treatment response significantly [166]. The CYP3A43 Ala340Ala genotype is strongly associated with biomarkers of vitamin D metabolism [35].

Many SNPs at the *CYP3A* locus show large differences in allele frequency across populations [167]. The CYP3A5*3 allele frequency ranges from ~50–55% in African Americans to 91% in Europeans [9,26]. The nonfunctional form is present in 85% of the Japanese, 65% of the Chinese, 67% of Mexicans, and 40% of American Indians [26]. Northern Europeans have signatures of positive selection in *CYP3A4* and *CYP3A43* promoter regions, which play a key part in drug metabolism [168]. Differences in genotype frequencies at loci of *CYP3A* subfamily genes between African American and white American populations have been described [163]. Genes *CYP3A4* and *CYP3A5* possess high diversity of haplotypes having different frequencies in different ethnic groups [5].

#### 2.4.3. CYP3A Inducers 

**Figure 4:**
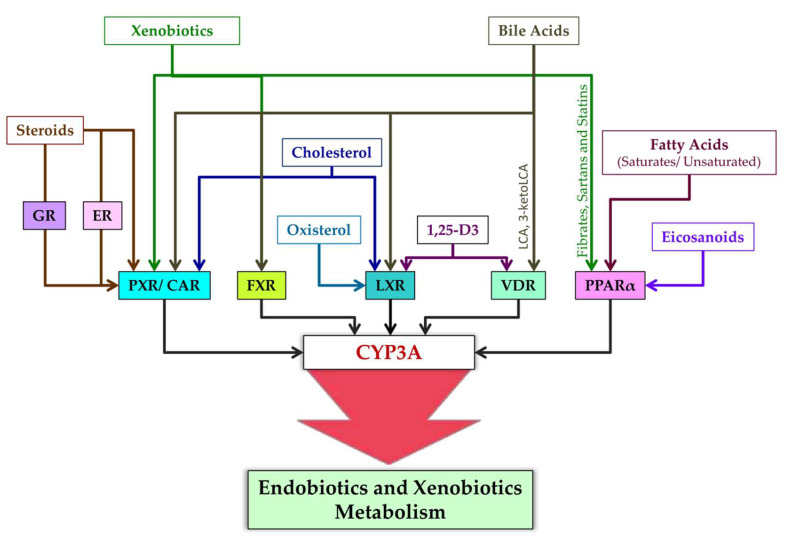
Ligands of nuclear receptors modulating CYP3A expression.

Ligands of nuclear receptors modulating CYP3A expression are presented in Figure 4. After binding to a ligand, PXR regulates the expression of genes whose products participate in cholesterol metabolism and bile acid metabolism [169]. Typical ligands of PXR are steroids [169]. These are diverse compounds including pregnanes, progesterones, corticosterones, testosterone, pregnenolone, bile acids, intermediate sterol compounds (such as 5-cholestanoic acid 3,7,12-triols, 7α-hydroxy-4-cholesten-3-one, and 4-cholesten-3-one), bile acid precursors, dexamethasone, and 17β-estradiol [76,93,170,171,172,173,174,175,176]. Endogenous activators of PXR also include metabolites of microbial origin, such as metabolites of bile acids and of tryptophan, e.g., indole-3-propionic acid [177].

CAR is constitutively active in the absence of ligands, takes part in physiological and pathophysiological pathways regulating glucose, lipid, and bile acid metabolism, and promotes cell proliferation, tissue regeneration, and cancer progression [178,179]. This constitutive activity, however, is inhibited by inverse agonist ligands [178]. Such endogenous ligands of CAR are testosterone metabolites androstanol and androstenol [180,181,182], estradiol [183], and cholesterol [184]. 

VDR is activated by 1,25-dihydroxyvitamin D_3_ [1,25(OH)_2_D_3_], which is an active metabolite of vitamin D and is involved in bone metabolism and maintenance of calcium-and-phosphorus homeostasis [176,185,186]. Furthermore, CYP11A1-produced hydroxyvitamin D derivatives 20S(OH)D_3_ and 20,23(OH)_2_D_3_ can act as “partial” or “biased” agonists (ligands) on VDR [187]. VDR is also engaged by bile acid metabolites: lithocholic acid (LCA) and 3-keto LCA [85,188].

FXR was originally identified as a receptor triggered by farnesol, which is an intermediate of cholesterol synthesis [189]. Later, bile acids were found to be endogenous ligands of FXR [190], and FXR target genes were identified that contribute to the modulation of bile acid biosynthesis, secretion, and reabsorption [191]. 

PPARs (all isoforms) are activated by fatty acids and their derivatives, for example, by eicosanoid and prostaglandin derivatives as products of lipoxygenase and cyclooxygenase pathways [192]. Saturated fatty acids have weaker binding affinity for PPAR, whereas polyunsaturated fatty acids have stronger affinity [193]. More specific ligands of PPARα are eicosanoids, 8(S)-hydroxyeicosatetraenoic acid, and leukotriene B_4_; of PPARγ are 15-deoxy-Δ12,14-prostaglandin, J2,15-hydroxyeicosatetraenoic acid, and 9- and 13-hydroxyoctadecadienoic acids; and of PPARβ/δ is 8(S)-hydroxyeicosatetraenoic acid [193]. PPAR activation is cell type specific, e.g., a ligand of PPAR called 15-keto-prostaglandin E_2_ is produced in lung and colon epithelial cells, whereas 15-deoxy-Δ12,14-prostaglandin J_2_ predominantly in macrophages [192,194]. Other ligands of PPAR are xenobiotic drugs fibrates, sartans, and statins [195,196].

Ligands of LXR are some endogenous compounds: cholesterol and the oxysterols (oxidized derivatives of cholesterol) called 22(R)-hydroxycholesterol and 24,25(S)-epoxycholesterol [193,197,198] as well as 1,25(OH)_2_D_3_ [199].

#### 2.4.4. CYP3A Inhibitors 

CYP3A enzymes metabolize a variety of compounds among which almost the only common feature is lipophilicity and relatively large size; therefore, it is not surprising that many of these compounds act as inhibitors of CYP3A enzymes [200]. 

For CYP3A enzymes, there are weak inhibitors, which increase the area under the concentration–time curve (AUC) by ≥1.25-fold to <2-fold, moderate inhibitors, which enlarge AUC by ≥2-fold to <5-fold, and strong inhibitors, which increase AUC by ≥5-fold. AUC is a graphical representation of a concentration or dose, of drug in plasma during certain period of time. The AUC gives insight into the bioavailability variables of the drug and its clearance rate from the body [201]. Strong inhibitors of CYP3A enzymes include such antifungals as itraconazole, ketoconazole, and voriconazole; anticancer agents ceritinib, idelalisib, ribociclib, and tucatinib; macrolide antibiotics clarithromycin and telithromycin; antivirals (used to treat HIV infection) indinavir, nelfinavir, ritonavir, and saquinavir; an atypical antidepressant called nefazodone; and the drug mibefradil for hypertension and angina pectoris [202,203].

Several mechanisms of inhibition of CYP3A enzymes are possible, as shown in in vitro studies, and one compound can act through several mechanisms [204,205]. Rapidly reversible inhibition is mediated by direct rapidly reversible binding of an inhibitor or its metabolite to an CYP3A enzyme, resulting in competitive or noncompetitive inhibition, the extent of which is determined by the enzyme’s relative binding constants of the inhibiting ligand (a substrate or an inhibitor), and by inhibitor concentration. The most potent reversible inhibitors of CYP3A enzymes include azole antifungals and first-generation inhibitors of the HIV protease [204].

N-alkyl–substituted compounds can often inhibit CYP3A enzymes reversibly, and in vitro, this effect increases after preincubation. The reason is the oxidation of the inhibitor giving rise to a nitrosoalkane, which forms a slowly reversible complex with the reduced heme in a CYP3A enzyme’s molecule. These compounds include macrolides, such as erythromycin; some antidepressants; and other drugs, e.g., diltiazem, lidocaine, and tamoxifen [204]. 

The mechanism of irreversible inhibition is thought to involve CYP3A-mediated formation of reactive metabolites that covalently bind to the enzyme thereby causing its inactivation. Irreversible inhibition of CYP3A enzymes is characteristic of some 17α-ethynyl–substituted steroids (ethinylestradiol and levonorgestrol), furanopyridine, an HIV protease inhibitor [204,206], and possibly other furans; for example, it is known that furanocoumarin—a component of grapefruit juice—can markedly inhibit the metabolism of substrates of CYP3A enzymes [12,16,207]. Naringenin is also considered as a CYP3A inhibitor of grapefruit juice but there are data that neither naringin nor naringenin are primarily responsible for this effect [208].

Owing to their nonspecific detergent effect, bile acids can suppress the activity of CYP3A enzymes [209,210]. 

In principle, knowledge about enzyme kinetics helps to extrapolate in vitro data to in vivo conditions, but such modeling requires the assumptions many of which are difficult to verify [211]. Furthermore, it is necessary to take into account the functioning of intestinal and hepatic CYP3A enzymes, the contribution of different isoforms to the formation of metabolites, and the fact that most inhibitors of CYP3A enzymes are also substrates of such an enzyme, and at a high turnover rate of an inhibitor, its rapid loss can take place [211,212]. For getting knowledge on ligand interactions with different CYP3A enzymes, recently it became possible to use the free available AlphaFold Protein Structure Database [213] containing three-dimensional structures of all CYP3A P450 enzymes from all organisms.

It should also be mentioned that CYP3A inducers usually affect the efficacy of co-administered drugs, whereas CYP3A inhibitors affect their safety; therefore, CYP3A inhibition is considered clinically more important [12]. 

## 3. Involvement of CYP3A Enzymes in Biological Processes

CYP3A enzymes have very broad substrate specificity and metabolize a wide range of compounds in terms of chemical and biological properties. They catalyze reactions of hydroxylation, N-demethylation, O-dealkylation, S-oxidation, deamination, and epoxidation [26] of endogenous and exogenous compounds. CYP3A perform physiological functions by taking part in such endogenous processes as steroid catabolism, bile acid metabolism, and lipid and vitamin D metabolism. CYP3A enzymes metabolize a wide variety of therapeutics and may play an important role in alterations of biological activities of drugs or in enhanced clearance of drugs as well as in drug interactions. For instance, CYP3A enzymes’ substrates are such endogenous compounds as hormones, cholesterol, bile acids, arachidonic acid, and vitamin D as well as the vast majority of drugs and of xenobiotics that are not pharmaceuticals [11,12,14].

### 3.1. Metabolism of Endogenous Compounds

#### 3.1.1. Biotransformation of Cholesterol and Bile Acids by CYP3A Enzymes

CYP3A enzymes are involved in cholesterol and bile acid metabolic pathways [214,215,216]. Cholesterol is metabolized to 4β-hydroxycholesterol [13] and to 25-hydroxycholesterol by CYP3A4 and CYP3A5 [13,217]. CYP3A4 has shown little contribution to cholesterol 22-, 24-, 26-, and 27-hydroxylation [218]. Excess cholesterol is converted to bile acids by various enzymes such as CYP7A1, CYP27A1, and CYP8B1, including CYP3A4 and CYP3A5.

In humans, most bile acid synthesis takes place in the liver via a pathway initiated by the rate-limiting enzyme cholesterol 7-hydroxylase (CYP7A1) and involving sterol 12-hydroxylase (CYP8B1) and sterol 27-hydroxylase (CYP27A1) [106,219,220,221]. Primary bile acids—cholic and chenodeoxycholic—are formed in the liver and are then converted in the intestine into secondary ones: deoxycholic and lithocholic [106,220].

CYP3A enzymes participate in bile acid biotransformation [214,215,216]. CYP3A enzymes are associated with a minor pathway for the biosynthesis of primary cholate of bile acids. 25-Hydroxylation of 5β-cholestan-3α,7α,12α-triol, which is an intermediate in the biosynthesis of cholic acid, is catalyzed by CYP3A4 and CYP3A5 [222]. In bile acid homeostasis, CYP3A4 catalyzes the 6α-hydroxylation of both taurochenodeoxycholic acid and LCA [223]. CYP3A activity is the main route of elimination of the toxic secondary metabolite of bile acids: LCA. This acid is metabolized to its 3-OH derivative by CYP3A4 and CYP3A5 [224]. Additionally, deoxycholic acid is metabolized into 1β-, 3β-, 4β-, 5β-, 6α-, 6β-, and 19-oxidized derivatives by CYP3A4 and CYP3A7 [215,216]. 

#### 3.1.2. Biotransformation of Hormones by CYP3A Enzymes

CYP3A enzymes play an important part in hormonal homeostasis. With regard to steroid hormones, the CYP3A subfamily plays an important role in the metabolism of androgens (testosterone, androstenedione, dehydroepiandrosterone, and dihydrotestosterone), progesterone, cortisol, and their metabolites [10,12,14]. CYP3A metabolizes testosterone, progesterone, and cortisol via the 6β-hydroxylation reaction unique to this P450 subfamily. The highest 6β-hydroxylation activity is observed in CYP3A4, followed by CYP3A5 and CYP3A7 [225,226]. In this context, testosterone stimulates progesterone 6β-hydroxylation, whereas progesterone inhibits CYP3A-mediated 6β-hydroxylation of testosterone [226]. Cortisone is also metabolized to 6β-hydroxycortisone by CYP3A enzymes [227,228]. 

2β-, 15β- and 16β-hydroxylation of testosterone is mediated by CYP3A4 [227,229]. The formation of 6β- and 2β-hydroxytestosterone is catalyzed by CYP3A5 and CYP3A7, while 2α-hydroxytestosterone generation is catalyzed only by CYP3A7 [228,230]. Extrahepatic CYP3A4 is more often responsible for the metabolism of hormones in situ, for example, it takes part in irreversible oxidation of testosterone in the prostate, thereby terminating its androgenic effect [14]. 

CYP3A4, CYP3A5, and CYP3A7 mediates 7β- and 16α-hydroxylation of dehydroepiandrosterone. The formation of 7β-hydroxydehydroepiandrosterone is catalyzed mainly by CYP3A4, and 16α-hydroxydehydroepiandrosterone by CYP3A7 [5,229,231]. Therefore, the profiles of metabolites of 16α- and 7β-hydroxylation of dehydroepiandrosterone can be useful for differentiation between CYP3A4 and CYP3A7 activities [231]. 

CYP3A7 performs a function in estriol synthesis [5]. Estradiol is metabolized by CYP3A4 giving rise to 2-, 4-, and 16β-hydroxylation products [232]. The formation of 2- and 4-hydroxylated estradiol metabolites is also mediated by CYP3A5 and CYP3A7, although their contribution is smaller than that of CYP3A4 [233]. In addition, CYP3A4, CYP3A5 and CYP3A7 metabolize estrone to form 2-, 4- and 16α-hydroxylated metabolites [234,235,236,237]. Progesterone is metabolized by CYP3A4 to form 2β-, 6β-, 16α- and 21-hydroxylated metabolites, as well as CYP3A5 and CYP3A7 to form 6β-hydroxyprogesterone [226,227]. 

#### 3.1.3. Biotransformation of Vitamin D by the CYP3A Subfamily

In the liver and small intestine, CYP3A4 and CYP3A5 are involved in vitamin D metabolism [25]. CYP3A4 contributes to 24-hydroxylation and 25-hydroxylation of vitamins D_3_. The active form of vitamin D—1,25-dihydroxycholecalciferol [1,25-(OH)_2_D_3_]—is a CYP3A4 substrate [14,85]. CYP3A4 participates in tissue-specific conversion of vitamin D_3_ metabolites to their respective inactive metabolites by performing the 24- or 25-hydroxylation of 1-hydroxyvitamin D_3_ and the 4β-hydroxylation of 25-hydroxyvitamin D_3_ [14]. CYP3A5 catalyzes 23- or 24-hydroxylation of 1,25-(OH)_2_D_3_ [238]. Although vitamin D is inactivated by other extrarenal hydroxylases such as CYP24A1, it is primarily biotransformed by CYP3A4.

#### 3.1.4. Biotransformation of Arachidonic Acid by CYP3A Enzymes

Arachidonic acid is the precursor to various physiologically active molecules such as epoxyeicosatrienoic acids (*EETs*), which are a class of functionally bioactive lipid mediators derived from the metabolism of long-chain polyunsaturated fatty acids under the action of multiple enzymes of three main families, including cyclooxygenases, lipoxygenases, and cytochromes P450 [239,240]. The role of eicosanoids—produced by cyclooxygenases and lipoxygenases—in the control of physiological and pathological processes is well known at present. CYP epoxygenase metabolites of arachidonic acid, i.e., *EETs*, act as lipid mediators inducing numerous biological responses in both cardiac and noncardiac tissues. *EETs* play an important part in blood pressure regulation, inflammation, and cell proliferation [239,240]. 

In the relevant human CYP-mediated pathway, the main CYP enzymes that form *EETs* are CYP2C8, CYP2C9, CYP1A2, and CYP3A4 in the liver [239,241] and CYP2J2 in the heart [242]. CYP4F2 is the main CYP subfamily that forms hydroxyeicosatetraenoic acids in the liver and kidneys [239,243]. CYP3A4 is involved in the biosynthesis of 5,6-, 8,9-, 11,12-, and 14,15-*EETs* in the liver [239]. Little is known about the participation of CYP3A5 and CYP3A7 in the production of *EETs* [11].

### 3.2. Biotransformation of Exogenous Compounds

The substrates of CYP3A enzymes are a wide range of prescription drugs as well as xenobiotics such as carcinogens benzo[*a*]pyrene, 7,8-dihydrodiol, and atlatoxin B [244,245]. In the lungs, CYP3A4 and CYP3A5 metabolize procarcinogens of tobacco smoke to active carcinogens, which are polycyclic aromatic hydrocarbons such as benzo(a)pyrene [246], whereas CYP3A4 participates in the activation of tobacco-specific carcinogen N′-nitrosonornicotine (NNN) in the liver [247].

CYP3A enzymes can metabolize many structurally and functionally distinct classes of drugs (Table 2) [74,248,249,250,251,252,253,254,255,256,257,258].

The involvement of CYP3A enzymes in the metabolism of most drugs underlies their main biomedical significance as enzymes that influence drug kinetics and as participants in drug interactions.

### 3.3. Endogenous and Exogenous Biomarkers of Activity of CYP3A Enzymes 

Accurate predictions of the activity of CYP3A enzymes are needed to provide effective pharmacotherapy to predict treatment outcomes or potential adverse effects of various therapeutic agents and to assess the development and progression of diseases.

Research is underway for identifying optimal endogenous biomarkers of the activity of CYP3A enzymes, e.g., derivatives of cholesterol, of bile acids, or of steroid hormones, which can be quantified by measuring the concentration of certain compounds in a urine or blood sample without the introduction of xenobiotics into the human body [259]. Appropriate combinations of such markers should make it possible to predict the activity of CYP3A enzymes while taking into account contributions of different isoforms [11]. For example, in clinical studies, the metabolic ratio of 6β-hydroxycortisol to cortisol in urine has already been characterized as an endogenous biomarker of CYP3A activities [260]. 

Many metabolites of endogenous compounds are used as markers of the activity of CYP3A enzymes both in vitro and in clinical studies [11,13,222,260,261,262,263,264]. The metabolic ratio of 1β-hydroxydeoxycholic acid to deoxycholic acid in urine has been proposed as a potential endogenous biomarker of CYP3A activities [214]. 19-Hydroxylated deoxycholic acid, which is specifically produced by CYP3A7, has been suggested as a marker of CYP3A7 activity in vitro, although the usefulness of this marker in vivo is unclear [263]. Profiles of 16α- and 7β-hydroxylation derivatives of dehydroepiandrosterone can be employed to discriminate the activities of CYP3A4 and CYP3A7, whereas the contribution of CYP3A5 to these products is negligible [231].

Furthermore, lately, plasma 4β-hydroxycholesterol—a cholesterol metabolite that is a substrate of CYP3A4 and CYP3A5—has been regarded as an endogenous biomarker of CYP3A activities. Although little is known about the isoform specificity of 6β-hydroxylation, the metabolic ratio of urinary 6β-hydroxycortisone to urinary cortisone has also been proposed as a biomarker for the activity of CYP3A enzymes [265], whereas the ratio of urinary 6β-hydroxycortisol to urinary cortisol (6β-OH-C/C) and plasma 4β-OH-cholesterol (4β-OH-C) is currently used to evaluate the activity of CYP3A enzymes [266,267]. In one study, a statistical predictive model was built by combining the ratios 7β-hydroxy-dehydroepiandrosterone/dehydroepiandrosterone, 16α-hydroxy-dehydroepiandrosterone/dehydroepiandrosterone, and 6β-hydroxy-cortisol/cortisol [264]. 

Predicting the activity of CYP3A enzymes by means of an exogenous compound is central to research on interactions between drugs that have similar metabolic pathways involving CYP enzymes or that are used simultaneously in the treatment of diseases [259,268]. Over the past few decades, a number of marker compounds have been used for this purpose. Among them, midazolam—a benzodiazepine derivative used in more than 70% of phenotyping cocktails—has become the “gold standard” [13,266,268]. This drug can be administered orally and intravenously for characterization of metabolism by CYP3A enzymes by means of its 1′-hydroxylation in the intestines and liver, respectively. It has a short half-life (based on excretion) allowing for the detection of acute metabolic changes, and it and possesses a favorable safety profile as well as a lack of transport by P-glycoprotein and other transporters [13]. Other substrates for phenotyping of the activity of CYP3A enzymes are alprazolam and nifedipine [269,270]. The activity assay of CYP3A enzymes in the liver by a 14C-erythromycin phenotypic breath test also use [271]. 

Among the problems with exogenous probes (e.g., midazolam and alfentanil), safety is often cited, especially for children and cancer patients. Nonetheless, phenotyping with midazolam can be carried out by means of a single dose or microdose to minimize adverse effects, whereas phenotyping with alfentanil can be performed by pupillary miosis measurement; in general, it is believed that the above-mentioned problem can be solved [13].

Some results of studies comparing exogenous with endogenous biomarkers of the activity of CYP3A enzymes are contradictory [260,272]. The lack of consensus is especially true for gender-related and ethnic differences in the activity of CYP3A enzymes [265,269]. Nevertheless, midazolam clearance appears to be superior to 4β-hydroxycholesterol in terms of the accuracy of predicting drug interactions. As a biomarker of CYP3A4, 4β-hydroxycholesterol is questionable because its concentration can be affected for example by the patient’s diet, by the presence of liver and kidney diseases, by the drugs used for their treatment, and by medications influencing cholesterol levels. Some authors believe that 4β-hydroxycholesterol is not suitable as a phenotypic marker of CYP3A activities [13].

## 4. CYP3A Involvement in Pathological Processes

### 4.1. Diseases Related to the Participation of CYP3A Enzymes in Bile Acid Metabolism

Bile acids are necessary for bile production and lipid absorption from the small intestine. They help with the excretion of cholesterol, hydrophobic metabolites of xenobiotics, toxins, and metals from the human body as well as play the role of signaling molecules in energy metabolism [74,106,221], participate in the regulation of lipid and glucose homeostasis, thermoregulation, and immune responses [221], and have anti-inflammatory properties under normal physiological conditions [107]. But the accumulation of large amounts of bile acids causes inflammation and damage to the liver [107]. High concentrations of bile acids are toxic to hepatocytes, enterocytes, and cholangiocytes and raise the risk of digestive-tract diseases, including cholestasis and cancers [74,106,273,274]. 

Bile acids undergo enterohepatic circulation: ~95% of their amount is reabsorbed and returned to the liver, but in each cycle they are synthesized de novo by hepatocytes to replace the bile acids that are eliminated [106]. The regulation of bile acid synthesis and metabolism is very complex and has to be tightly regulated to maintain nondangerous concentrations sufficient to perform a physiological function [107,221]. 

In humans, most bile acid synthesis occurs in the liver via a pathway initiated by the rate-limiting enzyme cholesterol 7-hydroxylase (CYP7A1) and involving sterol 12-hydroxylase (CYP8B1) and sterol 27-hydroxylase (CYP27A1) [106,107,219,220,221]. Primary bile acids—cholic and chenodeoxycholic—arise in the liver and are then converted in the intestine into secondary ones: deoxycholic and lithocholic [106,220].

The CYP3A subfamily participates in cholesterol and bile acid metabolic pathways: regulation of the conversion of cholesterol to 4β-hydroxycholesterol is performed by CYP3A4 and CYP3A5 [13], and bile acid biotransformation is performed by CYP3A enzymes [214,215,216]. Primary and secondary bile acids in turn alter the expression of CYP3A enzymes in the relevant organs: CYP3A4 and CYP3A5 in the liver and intestines and CYP3A7 and CYP3A43 only in the liver [220]. 

Cholestasis is a pathological condition where normal flow of bile is low or disturbed, and bile acids accumulate in the liver. The reason is either mechanical obstruction of bile ducts or defects in hepatic transporters [275]. The outcome is inflammation and damage to the bile ducts, followed by exposure of hepatocytes to high concentrations of bile acids, which can result in hepatocyte death [275,276]. For control over processes in the gastrointestinal tract in cholestatic conditions, the composition of bile acids and the size of their pool are important, and hydrophobic bile acids are especially cytotoxic [107,276]. 

In vitro and in vivo studies show a decrease in CYP3A activity in cholestatic liver disease. Nevertheless, CYP3A4 activity can be stimulated early in cholestasis because bile acids can induce CYP3A4 expression indirectly through FXR [76,277] and PXR [277,278,279]. Furthermore, LCA activates CYP3A4 mainly through VDR in the colon [85]. There are reports of early activation of CYP3A enzymes in animal models of cholestasis induced by bile duct ligation [278,280]. CYP3A subfamily activation as an in vivo adaptive response to cholestasis has been confirmed in experiments on humanized CYP3A4/lacZ transgenic mice carrying a genetic construct containing an upstream regulatory region of the human CYP3A4 gene [278]. In rat models of cholestasis, when cholestasis is moderate, mRNA and protein expression of CYP3A1 is significantly increased while the expression of CYP3A2 is unchanged; the expression and activity of both enzymes are sharply lower in severe cholestasis; CYP3A1 and CYP3A2 are the metabolically most important isoforms of CYP3A in male rats [280]. 

On the other hand, at later stages of the disease, CYP3A4 activity is downregulated due to elevated levels of estrogen and bile acids in the blood [74]. As the concentration of bile acids increases, the activity of several CYP enzymes in rats, e.g., CYP3A2, diminishes because bile acids exert nonspecific detergent effects on these enzymes [209]. As for estrogen, cholestasis is associated with its elevated levels (in mice) [281], whereas estrogen is involved in the regulation of CYP3A4 and suppresses its expression in humans [28]. 

The inhibitory effect of bile acids on the activity of CYP enzymes correlates with hydrophobicity of these acids. The most toxic and having a strong inhibitory effect is LCA; deoxycholic and chenodeoxycholic acids are less toxic, and cholic acid is the least toxic bile acid [74]. Among bile acids, LCA is the most active PXR ligand [221]. A protective role of PXR against bile acid toxicity is known: CYP7A1 expression is lower compared to a control in human hepatocytes treated with a PXR agonist: rifampicin [282]. 

The difference in the effects of early and late cholestasis on CYP3A enzymes’ activity is likely due to differences in the activation of PXR and CAR. It is known that PXR interacts with nuclear receptors CAR and FXR, which control homeostasis of cholesterol, bile acids, bilirubin, glucose, and lipids [283]. FXR is an important player in processes of bile acid detoxification. FXR activated by them induces CYP3A4 expression and phase I hydroxylation reactions [106,107]. In addition, bile acids activate a membrane bile acid receptor: TGR5. Activation of FXR and of a G protein–coupled bile acid receptor (GPBAR1, i.e., TGR5) by agonists improves insulin and glucose sensitivity and stimulates energy metabolism thus preventing diabetes mellitus, obesity, and nonalcoholic fatty liver disease, which is one of the leading causes of liver diseases, because of its association with obesity, type 2 diabetes mellitus, and dyslipidemia [107,141,284]. 

Nonalcoholic fatty liver disease is reported to correlate with reduced expression and function of CYP3A4 [141]. Enzymatic activity and mRNA expression of CYP3A4 are low in patients with nonalcoholic fatty liver disease, and this observation is confirmed by experiments on mice in vivo and on cultured cells in vitro [284,285,286,287,288,289]. There is evidence that changes in hepatic levels of some miRNAs, in particular miR-150-5p and miR-200a-3p [141] in nonalcoholic fatty liver disease can post-transcriptionally modulate the expression of CYP3A4 [140,290,291,292].

Cardiac dysfunction is associated with an increase in concentrations of bile acids in the blood during liver diseases. It is possible that bile acids as a ligand can activate FXR, which can play a part in the development of atherosclerosis [293,294].

FXR, CAR, and PXR have many regulatory effects in common, and their functions—in the context of regulation of liver detoxification and bile acid metabolism—overlap substantially. An increase in bile acid clearance can be caused by CYP3A4 upregulation after the stimulation of vitamin D receptor by vitamin D and LCA [221]. Clinically significant outcomes of cholestatic diseases (and of treatment with bile acids or their derivatives) may be affected by the activity of some drug-metabolizing CYP enzymes regulated by the same nuclear receptors as CYP3A enzymes are [221]. In theory, polymorphisms in CYP3A4 and PXR genes may affect the degree of bile acid detoxification [74], but evidence is not yet available. The function of PPARα as a key transcription factor in the regulation of bile acid metabolism and lipid metabolism is also important for proper liver functioning and for the pathogenesis of nonalcoholic fatty liver disease and nonalcoholic steatohepatitis [295,296].

Stimulation of CYP3A4 activity in cholestasis may be an effective therapeutic approach to such diseases [74]. In clinical practice, rifampicin and ursodeoxycholic acid are used to treat cholestasis, consistently with this acid’s ability to reduce the toxicity of more hydrophobic bile acids [74]. Ursodeoxycholic acid can induce CYP3A enzymes and reduce the concentration of their substrates [221]. 

It must be mentioned that several mechanisms of therapeutic action of ursodeoxycholic acid have been proposed [275]. Aside from inducing CYP3A enzymes, ursodeoxycholic acid can inhibit intestinal absorption of hydrophobic bile acids thereby raising the hydrophilicity of the bile acid pool [58,297]. Ursodeoxycholic acid also contributes to the restoration of the “bicarbonate umbrella”: biliary secretion of bicarbonate ions, which protect cholangiocytes from damage by bile acids [298]. Although the therapeutic benefits of ursodeoxycholic acid are well documented, many patients do not adequately respond to treatment with this compound, whereas high doses of ursodeoxycholic acid are associated with a high risk of esophageal and gastric varicose veins and a need for a liver transplant [275,299].

New therapeutic approaches are being developed for the treatment of cholestatic pathologies, including agonists of FXR, which mediates the induction of CYP3A4 expression by bile acids [106]. Furthermore, other derivatives of natural bile acids have been proposed: “bile mimetics,” such as nor-ursodeoxycholic acid and obeticholic acid, which is a potent FXR agonist [300].

### 4.2. Diseases Associated with the Participation of CYP3A Enzymes in Arachidonic Acid Metabolism

Arachidonic acid metabolites known as eicosanoids (*EETs*), a subclass of oxylipins, have a wide range of physiological effects in cardiovascular homeostasis and regulation of cell growth, inflammation, and immune responses. *EETs* function as regulators of cardiac, vascular [301,302,303,304], and renal physiology [305,306,307]. In the cardiovascular system, *EETs* serve as vascular relaxation factors independent of nitric oxide and prostacyclin I2 [308].

Eicosanoids (*EETs*), whose emergence depends on CYP3A-mediated metabolism, are implicated in the pathophysiology of various diseases. *EETs* promote the growth of a primary tumor and metastasis as well as the exit of a tumor from an indolent state [309]. The functions of *EETs* are associated with carcinogenesis, metastasis of various cancers (including colon, liver, kidney, breast, and prostate cancers [309,310,311,312,313]), with angiogenesis [314,315,316,317], and restoration of cardiac tissue after ischemic stroke [318].

The CYP3A4 epoxygenase, responsible for the production of *EETs*, is overexpressed in breast cancer and is linked with the initiation and progression of breast cancer [319]. CYP3A4 activity can accelerate tumor progression that is independent of activation of oncogenes. This notion is supported by the results of a study on breast cancer cell lines in which 8,9-, 11,12-, and 14,15-*EETs* are synthesized with the participation of highly enzymatically active CYP3A4 [313]. In that work, silencing of CYP3A4 blocked the cell cycle at the G2–M checkpoint and induced apoptosis of MCF7 cells by inhibiting STAT3 (Tyr-705) phosphorylation, thereby inhibiting the growth and survival of the tumor cells; a knockdown of CYP3A5 suppressed the proliferation of cell lines MCF7, T47D, and MDA-MB-231 to various degrees [313]. CYP3A4 takes part in breast cancer progression by stimulating angiogenesis through the activation of vascular endothelial growth factor (VEGF) [309,310,320] and in cancer cell proliferation through the activation of phosphatidylinositol 3-kinase (PI3K)–protein kinase B (AKT) and STAT3 pathways partially because of the synthesis of (+/−)-14,15-*EET* [313]. 

Mechanisms have been discovered by which CYP monooxygenases specific for cancer cells promote tumor progression and are associated with breast cancer mitochondria, whereas *EETs* stimulate the electron transport chain/respiration and inhibit AMPKα (AMP-activated protein kinase) [321]. A CYP3A4 knockdown or biguanide inhibition activates AMPKα, promotes autophagy, and prevents breast tumor formation [321]. These findings indicate that arachidonic acid, CYP epoxygenases metabolizing it, and *EETs* are connected with mitochondrial function and oxidative stress in cancer cells; this phenomenon may be another potential mechanism underlying antiapoptotic actions of these substances. In addition, *EETs* promote epithelial–mesenchymal transition (EMT) and drug resistance via STAT and AKT signaling pathways [313,322,323,324,325]. 

CYP3A4 is necessary for tumor formation in ER^+^HER2^−^ breast cancer because this enzyme suppresses autophagy, in part by inhibiting AMPK activation [326]. Although the production of an *EET* via CYP3A has been proven to correlate with the progression of breast cancer, little is known about the role of this enzyme in the development of chemoresistant breast cancer. A study on tamoxifen-resistant breast cancer [327] revealed that CYP3A4 expression and its epoxide product, 11,12-*EET*, are upregulated in tamoxifen-resistant MCF-7 cells (TAMR-MCF-7) compared to control MCF-7 cells. In that work, treatment of TAMR-MCF-7 cells with ketoconazole and azamulin (selective CYP3A4 inhibitors) or 14,15-epoxyeicosa-5(Z)-enoic acid (an antagonist of epoxyeicosatrienoic acid) inhibited cell proliferation and restored sensitivity to 4-hydroxy tamoxifen. It was found there that the molecular mechanism underlying this process is the suppression of E2F1-dependent transcription of the peptidyl prolyl isomerase gene (Pin1); it codes for a major regulator of angiogenesis and of characteristics of the epithelial–mesenchymal transition of TAMR-MCF-7 cells. Suppression of Pin1 via inhibition of *EETs* blocked proliferation and migration of TAMR-MCF-7 cells and angiogenesis mediated by these cells.

In human hepatoma cell line Hep3B, overexpression of CYP3A4 also promotes cell growth and cell cycle transition from the G1 to S phase; these effects are attenuated by a putative *EET* receptor antagonist: 14,15-epoxyeicosa-5(Z)-enoic acid [328]. 

*EETs* are known to exert anti-inflammatory actions in human and mouse tissues [329], thereby complicating the biological effects of *EETs* on cancer. Experimental studies suggest that 11(12)-*EET* can reduce the expression of TNF [329], whereas 14(15)-*EET* can downregulate TNF-α and IL-1β [330]. 

Arachidonic acid metabolism is reported to be one of the metabolic pathways most affected by SARS-CoV-2 infection [331]. EETs have been shown to take part in kidney damage [332]. Therefore, it seems that a therapeutic strategy based on specific inhibitors or inducers of CYP3A, depending on circumstances, may be useful against COVID-19.

### 4.3. Roles of CYP3A Enzymes in Diseases Associated with the CYP3A Involvement in the Metabolism of Sex Steroids 

The role of CYP3A enzymes in the metabolism of sex steroid hormones implies an association with the development of hormone-dependent diseases. It has long been known that CYP3A enzymes are expressed in normal and tumorous tissues of the breast [333,334,335] and of the prostate [336,337], in cells of the endometrium and cervix [338,339], and in ovarian tumors [340].

According to [341], the concentration of CYP3A4 in breast cancer tissue varies from 0.5 to 63 pmol/(mg protein). Overexpression of CYP3A4 in a cancerous tissue compared to the normal tissue in most cases correlates with predisposition to breast cancer [319,342,343] and ovarian cancer [344]. On the contrary, in prostate cancer, CYP3A4 is underexpressed [345,346]. 

CYP3A4 participates in the conversion of 17-β-estradiol to potentially genotoxic 16-α-hydroxyestrone. It has been demonstrated that high expression of CYP3A4/5 at mRNA and protein levels in tumor tissues of the mammary gland correlates with lymph node metastasis (lymph node status) and may be a key sign of an unfavorable prognosis of breast cancer. In this context, CYP3A4/5 expression is not associated with the presence of ER and progesterone receptor [342].

The CYP3A4 protein is expressed in both the tumor and surrounding normal breast tissue but is overexpressed in the tumor, according to data obtained by an immunohistochemical method in tissue samples from women with infiltrating ductal carcinoma; the staining was positive in all tumor samples but only in 68% of normal breast tissue samples [347]. Additionally, according to some data [319] obtained by immunohistochemistry, in the stroma and glandular tissue of a mammary gland containing a tumor, CYP3A4 is overexpressed as compared to the healthy tissue. By contrast, according to other data (obtained by immunoblotting), CYP3A4 expression is lower in breast tumor tissue compared to the morphologically normal adjacent tissue [348].

Immunohistochemical analysis of primary epithelial ovarian cancer tissue has revealed high protein levels of several P450 enzymes, namely CYP3A5, CYP3A7, and CYP3A43, as compared to normal ovarian tissues [344]. 

Androgen receptor-regulated signaling pathways play a key role in the progression of the prostate cancer in which androgen-metabolizing enzymes CYP3A4, CYP3A5, and CYP3A43 are expressed. Several CYP enzymes, including CYP3A43, can be considered prognostic and diagnostic markers of prostate cancer [349]. In particular, CYP3A hydroxylates testosterone, which is essential for the normal functioning of prostate cells and is crucial for the progression of prostate cancer, to less active metabolites. Measurement of mRNA levels of CYP3A genes in prostate samples has shown that basolateral cells of the prostate normally express CYP3A enzymes abundantly, but in the tumor tissue, mRNA and protein levels of CYP3A5 are markedly lower [346]. 

Similar results have been obtained by immunohistochemical analysis in surgical tissue samples from patients with prostate cancer. CYP3A4 immunoreactivity proved to be significantly lower in tumor tissues than in a benign epithelium; diminished immunoreactivity of CYP3A4 correlated with a poor prognosis of the disease [345]. In contrast, the authors of ref. [350] have demonstrated androgenic induction of *CYP3A5* mRNA in prostate cancer LNCaP cells.

PXR and CAR are overexpressed in tumor tissues, for example in prostate cancer [351], breast cancer [352], endometrial cancer [353], and ovarian cancer [354]. Various levels of expression of PXR, which regulates the expression of CYP3A genes, have been found in endometrial cancer tissues in contrast to normal tissues. As compared to tissues with PXR downregulation, tissues with high expression of PXR also featured overexpression of CYP3A4/7 and underexpression of estrogen receptor. Among endometrial cancer cell lines, HEC-1 cells, which overexpress PXR and underexpress ER and progesterone receptor, show a stronger transcriptional response of the PXR–CYP3A pathway to ligands of PXR [355]. Moreover, ligands of PXR enhance PXR-mediated transcription in a ligand- and promoter-dependent manner, resulting in differential regulation of the expression of PXR’s individual target genes, especially CYP3A4 and MDR1, in endometrial cancer cells [356]. These data suggest that steroid/xenobiotic metabolism in tumor tissue via the PXR–CYP3A pathway may play an important part in the pathogenesis of endometrial cancer [355,357]. 

Several miRNAs potentially regulated by CAR and capable of activating CYP3A4 were identified by [353]. The discovered miRNAs are tumor suppressors, and their level is low in malignant tumors of the endometrium.

Expression of PXR has been found in human prostate tumor tissues, and treatment of PC-3 cells with SR12813 (a selective agonist of PXR) stimulates the expression of multidrug resistance 1 (MDR1) and CYP3A4 and enhances the resistance of these prostate cancer cells to chemotherapy. In the normal prostate, the expression of PXR turned out to be the highest in the stratum basale; at earlier stages of the disease, PXR immunoreactivity is higher in prostate tumor tissues than in normal tissues, whereas with progression to later stages, the PXR expression tends to decline [351]. 

### 4.4. Diseases Related to the Participation of CYP3A Enzymes in Vitamin D Metabolism

Vitamin D has multiple effects on the biological processes that regulate the metabolism of calcium and phosphorus and also affects proliferation, differentiation, and apoptosis of cells as well as immune regulation. Epidemiological studies and experimental laboratory data show that vitamin D deficiency correlates with the onset and progression of many common chronic diseases such as bone metabolism disorders, tumors, cardiovascular diseases, infectious diseases, autoimmune diseases, diabetes mellitus, and others [358,359,360,361,362,363,364,365]. 

The proportion of people with vitamin D deficiency is high among patients with chronic infection caused by hepatitis C virus, non-alcoholic fatty liver disease, multiple sclerosis, psoriasis, osteoarthritis or chronic kidney disease [358]. A recent study showed vitamin D deficiency in 82% of hospitalized patients with COVID-19 [366]. CYP3A4, by taking part in the inactivation of an active vitamin D metabolite [1.25(OH)_2_D_3_], may have a significant impact on circulating vitamin D levels and hence calcium homeostasis, which in turn may influence bone and immune-system health [367,368] by downregulating the overexpression of inflammatory cytokines such as IL-1α, IL-1β, and TNF [369,370]. Pathologies such as vitamin D–dependent rickets and osteoporosis can progress because of the inactivating effect of CYP3A4 on the active form of vitamin D [361,365,371].

It is possible that the progression of cardiovascular diseases may be due to the inactivating effect of CYP3A4 on the active form of vitamin D because a low concentration of 1,25(OH)_2_D_3_ in blood serum is strongly associated with the initiation of cardiovascular diseases [372] and with the incidence of arterial hypertension [373]. 

Genetic factors have been recognized as some of the main causes of vitamin D deficiency [374]. For instance, in patients with rickets, there is a decreased serum concentration of vitamin D metabolites owing to mutations in CYP3A4; in particular, the CYP3A4 I301T mutation results in a stronger activity inactivating vitamin D metabolites, thereby reducing levels of active vitamin D [375]; in other words, CYP3A4 polymorphisms can worsen some pathological conditions. Numerous studies indicate that the risk and prevalence of type 2 diabetes mellitus correlate with vitamin D deficiency [374,376,377,378,379].

Recent research articles have elucidated the relation between CYP3A4 polymorphisms and type 2 diabetes mellitus by showing that people with some CYP3A4 polymorphisms are at a higher risk of type 2 diabetes mellitus [380]. Nonetheless, regarding the possible association of CYP3A4 with diabetes mellitus, experimental clinical data are inconsistent. For example, it has been reported that the functional activity and expression of CYP3A4 are higher in experimental diabetes [381]. Fatty acids (palmitic, oleic, stearic, and linoleic) participate in the mechanism of CYP3A4 induction in experimental diabetes. It is these acids, not insulin, that raise the activity and mRNA and protein expression of CYP3A4, as demonstrated in vitro on HepG2 and Fa2N-4 cells that are incubated with the serum of rats with streptozotocin-induced diabetes [381]. 

During investigation into the expression of hepatic CYP enzymes in patients with diabetes mellitus, the results revealed that this disease correlates with a significant decrease in hepatic CYP3A4 enzymatic activity and protein levels. For instance, after an assay of the activity of seven major CYP enzymes in 38 patients with type 2 diabetes mellitus and 35 patients without diabetes (oral administration of the midazolam as a probe drug), it turned out that the activity of CYP3A enzymes in patients with diabetes mellitus is lower by approximately 38% [382]. It is also reported that hydroxylation of marker substrates—midazolam (at the 1′- or 4-position) and testosterone (6β-hydroxylation)—by CYP3A enzymes is notably reduced in liver microsomes of diabetic patients, regardless of the genotype of CYP3A genes [383]. A significant decrease in the activity and protein level of CYP3A4 has been registered in diabetic patients with nonalcoholic fatty degeneration of the liver or nonalcoholic steatohepatitis. Furthermore, this decrease in activity continued with growing disease severity as it progressed from nonalcoholic fatty degeneration of the liver to nonalcoholic steatohepatitis [289].

The ability of CYP3A4 to catalyze the inactivation of 1,25(OH)_2_D_3_ and of other active forms of vitamin D has an influence on cancer cell proliferation and may be clinically important in various common cancers, mostly breast, prostate, and colorectal malignant tumors [362,363,384,385,386]. A possible reason is that vitamin D takes part in cell proliferation by affecting the expression of genes p21 and p27, by promoting apoptosis and autophagy, by regulating angiogenesis, by upregulating antioxidants, and by exerting anti-inflammatory actions via downregulation of genes for multiple inflammatory mediators (interleukins; e.g., IL-12, IL-2, TNF, and interferon gamma) as well as by regulating various inflammatory cascades such as the NF-κB (nuclear factor kappa light chain enhancer of activated B cells) pathway, mitogen-activated protein kinase (MAPK) signal transduction, and cyclooxygenase-2 (COX-2) signaling [362]. 

An in vitro analysis of human liver microsomes has revealed the ability of CYP3A4 to inactivate other active forms of vitamin D, its 20(OH)D_3_ derivative, and other relevant metabolites that may also have a physiological effect on cancer cell proliferation [386]. There are also abundant data from various studies indicating that vitamin D reduces the risk of developing of numerous types of cancer, including lung, ovarian, prostate, breast, colon, non-Hodgkin’s lymphoma [371]. There is also convincing evidence that vitamin D reduces the risk of autoimmune diseases, such as multiple sclerosis and type 1 diabetes. Less convincing evidences exist for reducing the risk of type 2 diabetes, rheumatoid arthritis, osteoarthritis, hypertension and stroke [365,371,383].

### 4.5. Changes in the Expression and Activity of CYP3A Enzymes in Various Pathological Conditions

It is now clear that the expression and activity of CYP enzymes are affected by such pathological conditions as infection, inflammation, and cancer [10,387,388]. 

#### 4.5.1. Inflammation-Dependent Changes in the Expression and Activity of CYP3A Enzymes 

The influence of inflammation on the expression of CYP3A enzymes is an important topic because an alteration of these enzymatic activities leads to a change in pharmacokinetics of prescription drugs. Inflammation is a common sign of many diseases and is implicated in the pathogenesis of such illnesses as infectious diseases, cancer, diabetes mellitus, rheumatoid arthritis, and inflammatory bowel disease as well as age-related processes such as normal aging and metabolic aberrations [389]. Sources of inflammation are infections, e.g., hepatitis, human immunodeficiency virus (HIV) infection, and COVID-19 [390]. Aside from infections, other possible sources of inflammation in the human body are diseases of organs (the kidneys, liver, lungs, and heart), diabetes mellitus, autoimmune diseases (ankylosing spondylitis, psoriasis, systemic lupus erythematosus, Crohn’s disease, and celiac disease), and cancer. Additionally, possible sources of inflammation are vaccination, a surgical procedure, a critical condition of a patient, and treatment with immunomodulatory agents, anti-TNF antibodies, or monoclonal antibodies [390]. 

In such disease states, the regulation of CYP enzymes is connected with inflammation status [389]. It is recognized that changes related to CYP enzymes are a common consequence of the immunostimulation following infection and inflammation [391,392]. The source of modulation of CYP enzymes’ activities is endogenous inflammation markers: cytokines, adipokines, lipid metabolites of nitric oxide, proteases, and reactive oxygen species [132]. Inflammation is accompanied by suppression of the CYP enzymes that metabolize xenobiotics, including medical drugs [393]. The most studied CYP subfamily in inflammation is CYP3A [390].

In rodent and cell models, transcription of CYP genes is reported to be downregulated by inflammatory signals, e.g., cytokines. Such studies have shown that IL-1β, IL-1α, *IL*-6, and TNF indirectly lower the activity of multiple P450 enzymes [129,394]. Several clinical studies have revealed low activity of CYP3A4 in inflammatory conditions [271,395]. Some clinical research indicates that inhibitors of IL-6 enhance drug metabolism via CYP3A4, implying that IL-6 is an important regulator of this enzyme [396]. It has been demonstrated that CYP3A4 activity is suppressed in adenovirus-infected primary hepatocytes and that adenovirus-induced modification of PXR expression may be responsible for the alterations of CYP3A4 activity in the liver [397]. 

##### Infections

In patients without a clinically significant liver disease, investigation into the effect of chronic hepatitis C on pharmacokinetics of a probe of CYP3A enzymes called midazolam has uncovered weaker CYP3A4 activity in patients with chronic hepatitis C than in healthy volunteers [398,399]. In HIV-infected patients, a study on the activity of CYP enzymes—as assessed by phenotypic tests based on caffeine, dextromethorphan, and midazolam—has revealed a lower activity of CYP3A4 (18%) in HIV-infected adults compared with a control group [400]. Another study involving phenotypic tests based on midazolam, dextromethorphan, and digoxin showed that the activity of CYP3A enzymes is approximately 50% lower in HIV-infected patients than in healthy volunteers [401].

The most vivid pathophysiological feature of COVID-19 is the state of an excessive inflammatory response that may affect the expression of CYP enzymes [402]. The impact of a release of immunogenic proteins in COVID-19 on the activity of CYP enzymes is still largely unexplored. The first research article in this field addressed the effect of moderate-to-severe COVID-19 on enzymatic activity of six major CYP proteins in patients with SARS-CoV-2 infection by a phenotyping-cocktail method [403]. In that report, the activity of CYP enzymes and subsequent phenotypic classification were based on metabolic ratios, and evaluation of the metabolic ratios uncovered a decrease in the activity of CYP3A enzymes by 22.8%. Mean serum levels of C-reactive protein, IL-6, and TNF were significantly higher during SARS-CoV-2 infection than at 3 months after the illness [403]. 

Furthermore, research has been conducted on the influence of COVID-19 on substrates of CYP enzymes [404]. For instance, it has been documented that plasma concentrations of some substrates of CYP3A enzymes (lopinavir, darunavir, and direct oral anticoagulants) are significantly higher in patients with COVID-19 [405,406,407,408]. In particular, lopinavir concentrations correlated with levels of C-reactive protein and IL-6 because these concentrations diminished after administration of an anti–IL-6 antibody (tocilizumab) [407,409].

It has also been reported that CYP3A enzymes can have an effect on COVID-19 pathophysiology through arachidonic acid metabolites and vitamin D. For example, arachidonic acid metabolism is one of the most affected metabolic pathways during SARS-CoV-2 infection [331], and *EETs* (whose production is catalyzed by CYP3A enzymes) play a role in kidney damage in this context [332]. The level of an active form of vitamin D, which attenuates overexpression of such inflammatory cytokines as IL-1α, IL-1β, and TNF [369], is determined by CYP3A4 activity. Serum vitamin D levels are low in most of critically ill COVID-19 patients [410]. Thus, CYP3A4, by participating in the metabolism of vitamin D and eicosanoids, may be implicated in COVID-19 pathogenesis. On the other hand, because the expression of CYP3A enzymes can be significantly altered by inflammatory factors in patients with COVID-19, drug pharmacokinetics may be differ too among these patients.

##### Inflammatory Conditions

There are several relevant articles about patients with metabolic disorders. Research papers about the liver tissue of patients with diabetes mellitus point to low expression and activity of CYP3A enzymes as compared to controls [383]. There is evidence of reduced clearance of lidocaine (primarily metabolized by CYP3A enzymes) in patients with type 1 diabetes mellitus [411]. 

Type 2 diabetes mellitus is associated with upregulation of inflammation markers such as IL-6 and TNF. High levels of IL-6 and TNF correlate with suppression of some drug-metabolizing enzymes, especially isoforms of CYP3A proteins [412]. A recent study based on a test involving a cocktail of probe drugs (including midazolam) for CYP3A enzymes revealed an approximately 38% decline of the mean metabolic activity of CYP3A enzymes in patients with type 2 diabetes mellitus in comparison with controls [382]. In that report, IL-6 concentration significantly negatively correlated with the activity of CYP3A enzymes in a univariate analysis of data from all diabetic and nondiabetic patients. These results suggest that the deficient CYP-mediated clearance of drugs in type 2 diabetes mellitus may be related to inflammatory processes.

Research on a wide range of cytokines in patients with rheumatoid arthritis in relation to the phenotype of CYP3A4—quantified as the concentration of an endogenous metabolite (4β-hydroxycholesterol) in the serum of patients treated with inhibitors of TNF—has detected significant associations between cytokine levels and the CYP3A4 phenotype. The activity of CYP3A4 has been shown to correlate with levels of proinflammatory cytokines, in particular IL-1Ra (IL-1 receptor antagonist), IL-6, and C-X-C motif chemokine ligand 8 (CXCL8), and to negatively correlate with the CYP3A4 phenotype during the treatment with inhibitors of TNF. These findings indicate that the immune response linked with elevated levels of IL-1Ra, IL-6, and CXCL8 can suppress CYP3A4-mediated metabolism [413]. In another work, patients with rheumatoid arthritis also manifested lower plasma concentrations of 4-β-hydroxycholesterol (an endogenous metabolite of CYP3A4) than did healthy controls [414]. The role of IL-6 in the diminished CYP3A-mediated clearance of drugs in rheumatoid arthritis has been documented in clinical trials of monoclonal antibodies targeting IL-6 [415,416,417]. These studies have shown that pharmacological inhibition of IL-6 restores the activity of CYP3A enzymes and significantly reduces the therapeutic action of simvastatin.

Ulcerative colitis and Crohn‘s disease are two widespread types of inflammatory bowel diseases that are characterized by chronic and progressive inflammation in intestines, e.g., in the colon. In colon biopsy samples from patients with ulcerative colitis and patients with Crohn’s disease, a DNA microarray analysis has uncovered broad suppression of genes involved in drug metabolism; this dysregulation is accompanied by pronounced downregulation of PXR [418]. Some authors have compared serum levels of proinflammatory cytokines and pharmacokinetic parameters of drugs–substrates of CYP3A enzymes between healthy individuals and patients with ulcerative colitis or Crohn’s disease [419,420]; it has been demonstrated there that higher baseline serum levels of proinflammatory cytokines (TNF, IL-1β, IL-6, and IL-8) in the patients do not cause a change in CYP3A4 activity, as evidenced by the pharmacokinetic parameters of the drugs–substrates of CYP3A enzymes in such patients. Those researchers concluded that inflammatory bowel disease does not elevate the production of proinflammatory cytokines to the clinically significant levels that could alter pharmacokinetics of drugs–substrates of CYP3A enzymes [419,420]. Nonetheless, examination of intestinal biopsy samples from people with and without Crohn’s disease and quantification of CYP3A4 expression by Western blotting indicate a significant decrease in protein expression of CYP3A4 in the ileum (by 45%) and colon (by 78%) in subjects with Crohn’s disease relative to subjects without it [421]. 

In biopsy samples, investigators have demonstrated a decrease in *CYP3A4* and *PXR* mRNA expression in inflamed small-intestinal tissue versus noninflamed duodenum within individual subjects (children) with Crohn’s disease but not in controls [422]. Significant underexpression of CYP3A proteins has been confirmed in the duodenum during active inflammation in children with Crohn’s disease [423]. Research into hepatic and intestinal CYP3A4 activities by means of intravenous and oral midazolam and budesonide in patients with Crohn’s disease and celiac disease has detected a marked drop of CYP3A4 activity in vivo in patients with celiac disease [424]. One of the limitations of that study is a relatively small sample size, which makes the results less generalizable. The findings about downregulation of CYP3A4 mRNA in the intestine in celiac disease has been reproduced in biopsy samples from the duodenum and ileum [425].

##### Cancer

Inflammatory responses play a crucial part in various stages of carcinogenesis, including initiation, promotion, malignant transformation, invasion, and metastasis. Immune cells that infiltrate tumors participate in extensive and dynamic interactions and antagonistic and synergistic effects with cancer cells. Components of cancerous inflammation include the chemokines, prostaglandins, and cytokines that indirectly suppress the activity of CYP enzymes [426]. In an activity assay of CYP3A enzymes in the liver by a 14C-erythromycin phenotypic breath test in cancer patients with a C-reactive-protein level >10 mg/L, a decline of CYP3A enzymes’ activity by 30% was recorded as compared with patients without the acute phase response [271]. Analysis of phenotypic activity of CYP3A enzymes (the omeprazole sulfone/omeprazole ratio) in advanced ovarian cancer suggests that reduced CYP3A activity correlates with elevated serum concentrations of C-reactive protein, IL-6, IL-8, and TNF [427]. In patients experiencing cancer progression, the expression and activity of CYP3A4 is significantly lower, which can be explained by a greater plasma concentration of inflammation mediators, in particular, IL-6 [428,429]. 

The course of inflammatory responses can be influenced by such CYP3A substrates as bile acids because the latter interact with certain receptors, e.g., FXR, VDR, and LXR [293]. These receptors are expressed in immune cells such as monocytes, macrophages, natural killer cells, dendritic cells, T- and B-lymphocytes [426]. For instance, a launch of the SHP–FXR pathway blocks the JNK cascade and prevents the binding of NF-κB to promoters of genes encoding proinflammatory cytokines. FXR stimulates the binding of nuclear receptor corepressor 1 (NCOR1) to promoters of proinflammatory genes, thereby hindering the interaction of NF-κB with them [430]. The VDR signaling pathway performs an important function in the regulation of inflammatory responses—especially in inflammatory bowel disease—by transmitting signals of bile acids via VDR within the adaptive immune system [431,432]. LXR is relevant and important for macrophage biology in the context of atherosclerosis, which is currently regarded as a chronic inflammatory disease [433].

#### 4.5.2. Aberrant Intratumoral Expression of CYP3A Enzymes

Overexpression of CYP3A4 in a malignant tissue compared to a respective normal tissue is usually associated with predisposition to breast [319,342,343] and ovarian [344] cancers and may play an important role in the initiation of endometrial cancer [355,357]. We already mentioned that CYP3A4 participates in breast cancer progression by stimulating angiogenesis through VEGF activation [309,310,320] and by promoting cancer cell proliferation through the activation of PI3 kinase–AKT and STAT3 pathways, in part via the synthesis of (+/−)-14,15-EET [313]. CYP3A4 is also required for tumor formation in ER^+^HER2^−^ breast cancer because this enzyme suppresses autophagy, in part by inhibiting AMPK (AMP-activated protein kinase) activation [326]. 

Higher expression of CYP3A4 and CYP3A5 than in adjacent normal tissues has been reported in studies on rhabdomyosarcoma, which is the most common soft tissue sarcoma in children [434]. In tumor samples in that study, mRNA expression levels of CYP3A family members proved to be elevated compared to the corresponding normal neighboring-tissue samples; similar differences in protein levels were found, too.

In another research article, analysis of *CYP3A4* and *CYP3A5* expression in tumor tissue of children with nonrhabdomyosarcoma soft tissue sarcomas also showed that in such patients, mRNA and protein expression levels of these genes are significantly higher in the tumor tissue than in nonmalignant neighboring tissue; estimated 5-year relative survival of the treated patients is ~50% because they typically present with tumor progression, recurrence, metastasis, and/or resistance to chemotherapy [435]. It has been demonstrated that CYP3A4 overexpression may correlate with metastasis of Ewing’s sarcoma [436].

The main clinical consequences of aberrant intratumoral expression of CYP3A4 may be mediated by administration of anticancer drugs that are CYP3A substrates during the treatment of Ewing’s sarcoma. Local expression of CYP3A enzymes in malignant tissue may contribute to the development of multidrug resistance or toxicity observed in this type of tumor. 

Recent investigation into the aberrant expression of CYP3A5 in cancer, depending on the malignancy and ability to metastasize, uncovered a role of CYP3A5 in cancer progression, metastasis, and invasion.

Data from early studies pointed to underexpression of CYP3A4 and/or -5 by a tumorous tissue in primary and secondary liver tumors. A study by R. Tsunedomi revealed that CYP3A5 expression is dramatically reduced during venous invasion in patients with hepatitis C–associated hepatocellular carcinoma (hepatitis C–associated HCC) [437].

In contrast to CYP3A4, aberrantly low CYP3A5 expression in HCC and a negative association between CYP3A5 expression and HCC malignant characteristics have been detected in a large HCC cohort; several reports show that CYP3A5 can act as an HCC suppressor and can counteract the malignant tumor phenotype [438,439].

For instance, CYP3A5 expression is often low in tumor tissues at the level of transcripts and proteins in hepatocellular hepatoma; in particular, this underexpression contributes to worse overall survival of patients and to tumor metastasis [438,439]. Ref. [438] indicates that CYP3A5 expression negatively correlates with several malignant characteristics and poor prognosis of HCC; e.g., lower levels of CYP3A5 are associated with more aggressive vascular invasion, poor differentiation, shorter time to disease recurrence after treatment, and worse overall survival of the patients. In the same study, it was found that CYP3A5 can produce reactive oxygen species (ROS) when it metabolizes its substrates, and ROS are known to serve as cellular secondary messengers activating or inhibiting signaling cascades. There is evidence that forced expression of CYP3A5 dramatically suppresses migration and invasion of HCC cells in vitro via inhibition of ROS–mTORC2–p-AKT signaling, and consequently CYP3A5-induced ROS accumulation is a key regulator of the activity of mTORC2: a serine/threonine kinase that translates external stimuli into processes related to cell growth. The reason is that CYP3A5 selectively inhibits the phosphorylation of kinase AKT at Ser473, thus blocking its activity [438].

While examining CYP3A5 expression in many cancers, the authors of ref. [440] discovered that CYP3A5 is aberrantly underexpressed in lung cancer. They demonstrated by RT-PCR, Western blotting, and immunohistochemistry that CYP3A5 is usually downregulated in lung adenocarcinoma (LUAD) tissues and cell lines. Low expression of CYP3A5 is significantly associated with poor prognosis among patients with LUAD. External stimulation of CYP3A5 expression significantly inhibits LUAD cell migration and invasion in vitro and dramatically suppresses metastatic capacity in vivo. A subsequent study has revealed that CYP3A5 significantly reduces the phosphorylation of a TGF-β signaling protein called SMAD1 in LUAD cells [440]. 

SMAD1 is a central mediator of TGF-β signaling and is involved in a wide range of biological activities including cell growth, apoptosis, development, and immune responses. Several research articles highlight an important stimulatory effect of SMAD1 on tumor cell invasion and metastasis in various types of cancer [441].

It has been shown that a decrease in SMAD1 phosphorylation in LUAD cells leads to the suppression of LUAD metastasis by means of the ATOH8–SMAD1 axis, namely, CYP3A5 interacts with transcription factor ATOH8, and this interaction in turn mediates SMAD1 pathway inactivation [440].

CYP3A5 is the main extrahepatic isoform of CYP3A expressed in the prostate. Androgen receptor (AR) signal transduction is crucial for the growth and progression of prostate cancer. Intratumoral activation of CYP3A5 in prostate cancer is reported to mediate the growth of prostate cancer cells by facilitating nuclear translocation of AR [442]. The observed influence on AR signaling is due to the change in CYP3A5 activity, not in CYP3A4 activity [443].

Thus, the mechanism of regulation of AR expression by CYP3A5 is related to the fact that CYP3A5 is a component of the feedback loop that modulates AR sensitivity to androgens. A decrease in the amount of AR in the nucleus as a result of inhibition of CYP3A5 causes suppression of growth in LNCaP and C4-2 cell lines. On the contrary, the CYP3A inducer rifampicin enhances the nuclear localization of AR. Accordingly, CYP3A5 regulates the translocation of AR into the nucleus and downstream signaling, which leads to tumor [442].

Proof that CYP3A5 inducers promote AR nuclear translocation, downstream signaling, and cell growth whereas CYP3A5 inhibitors reverse these actions is also offered by a study on African Americans, who often carry wild-type *CYP3A5* and overexpress the CYP3A5 protein. The observed alterations of AR activity turned out to be specific to changes in CYP3A5 activity because the effects were attenuated by a CYP3A5 knockout in MDAPCa2b cells [443]. 

Thus, taken together, the presented studies provide a new insight into the participation of CYP3A5 in carcinogenesis. Firstly, CYP3A5 has a protective effect against HCC progression by acting as a suppressor of the pathogenesis and metastasis; secondly, prometastatic signal transduction in LUAD depends on CYP3A5; and finally, CYP3A5 promotes AR nuclear translocation in prostate cancer thereby playing a crucial role in AR signaling. Table 3 summarizes information about the role of CYP3A in the conditions of the diseases. 

## 5. Concluding Remarks

**Figure 5:**
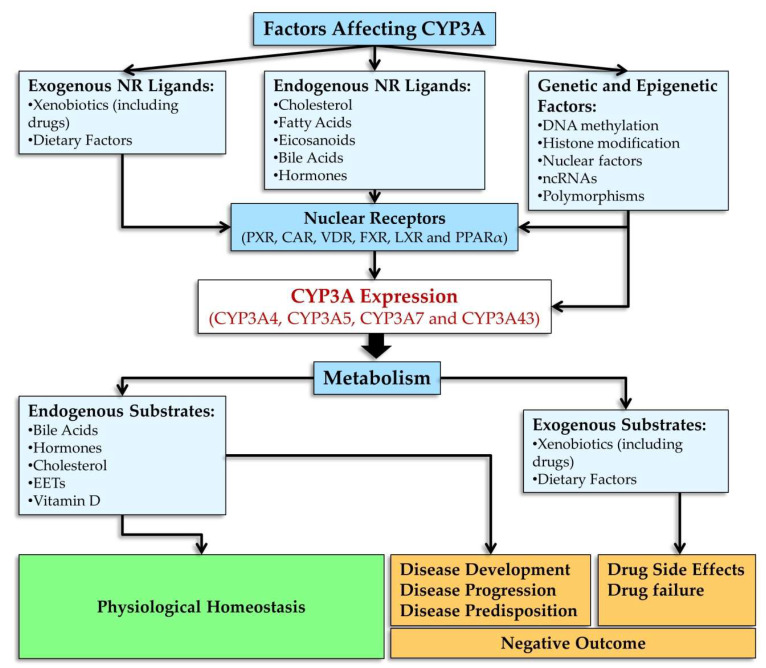
Effects of CYP3A enzymes on the metabolism of endo- and xenobiotics have an influence on a wide range of physiological and pathophysiological processes in the body.

This review is aimed at highlighting the main roles of CYP3A enzymes along with their unique characteristics in the metabolism of biologically active endogenous compounds and numerous xenobiotics that are important in clinical pharmacology as well as the involvement of these enzymes in a wide range of physiological and pathological phenomena. The scientific literature cited in this review attests to remarkable efforts and advances in the understanding how the CYP3A family of phase I biotransformation enzymes is integrated into the vast and complex network of physiological processes detoxifying endo- and xenobiotics. The function of CYP3A enzymes is complex because the effects of activation their genes are determined by a wide range of endogenous and exogenous ligands and by a unique regulatory system that involves CYP3A enzymes in many physiological and pathological processes in cells and tissues of the body (Figure 5).

The totality of evidence indicates that the activation of *CYP3A* genes can be either beneficial or detrimental during diseases of various organs and tissues. The ultimate effects depend both on the context of a disease and on the nature of ligands of the nuclear receptors that control *CYP3A* genes’ transcription.

Currently, the molecular mechanisms by which CYP3A enzymes take part in pathogenesis are well understood only for a few diseases; in particular, a role of CYP3A5 in carcinogenesis has been demonstrated. There are more reports of (i) diseases associated with the participation of CYP3A enzymes in the metabolism of endogenous compounds and (ii) pathological conditions affecting the expression and activity of CYP3A enzymes. The consequence of an alteration of these enzymes’ activities is a change in the pharmacokinetics of the drugs used for treatment. Much basic research has been conducted on the role of CYP3A enzymes in pathological processes, but clinical studies that are aimed at influencing the mechanisms of signaling pathways regulating *CYP3A* genes in various diseases are still insufficient, and further investigation is needed.

## Figures and Tables

**Table 2:** Classes of drugs that are metabolized by CYP3A enzymes.

	Drugs
Analgesics	acetaminophen, lidocaine
Anesthetic opioids	alfentanil
Antiarrhythmics	amiodarone, quinidine
Antibiotics	erythromycin
Anticancer agents	cyclophosphamide, epipodophyllotoxins, ifosfamide, toremifene, vincristine, vinblastine, vindesine, paclitaxel, docetaxel, cabazitaxel,
Anticonvulsants	clonazepam, trimethadione, zonisamide
Antidepressants	sirolimus, imipramine
Antiepileptics	carbamazepine, phenobarbital
Antihistamine drugs	fexofenadine, terfenadine
Antihypertensive drugs	nifedipine
Antitussive agents	dextromethorphan, codeine
Azole antifungals	fluconazole, itraconazole, ketoconazole
Immunosuppressants	tacrolimus, rapamycin, cyclosporine, FK506
Calcium channel blockers	diltiazem
Cholesterol-lowering drugs	HMG-CoA reductase inhibitors
Narcotics	cocaine
Nonsteroidal antiandrogens	flutamide
Nonsteroidal anti-estrogens	tamoxifen
Antiretrovirals (against HIV/AIDS)	amprenavir ritonavir
Psychoactive drugs	benzodiazepine, midazolam
Steroids	estradiol, testosterone, hydrocortisone
Tyrosine kinase inhibitors	lapatinib, dasatinib, erlotinib, imatinib

**Table 3:** CYP3A Involvement in Pathological Processes.

The Relationship of Diseases and CYP3A		Diseases	CYP3A	Possible Mechanism of CYP3A Participation in Pathogenesis	Ref.
Diseases Related to the Participation of CYP3A Enzymes in Bile Acid Metabolism		Cholestasis at early stages	CYP3A4 induction	FXR and PXR pathways	[76,277]
		Cholestasis at later stages	CYP3A4 activity downregulation	Elevated levels of estrogen and bile acids in the blood	[74]
		Nonalcoholic fatty liver disease	CYP3A4 reduction of expression and function	Possible post-transcriptionally modulation miR-150-5p and miR-200a-3p	[140,290,291,292]
Diseases Associated with the Participation of CYP3A Enzymes in Arachidonic Acid Metabolism		Breast cancer	CYP3A4 overexpression	Stimulation of angiogenesis through the activation VEGF. Proliferation in cancer cell through the activation of PI3K—AKT and STAT3 pathways	[309,310,320]
		ER^+^HER2^−^ breast cancer	CYP3A4 necessary for tumor formation	Suppression of autophagy, in part by inhibition of AMPK activation	[326]
		COVID-19	CYP3A activity	EETs production is catalyzed by CYP3A enzymes EETs play a role in kidney damage	[332]
Diseases Associated with the CYP3A Involvement in the Metabolism of Sex Steroids		Breast cancer	CYP3A4/3A5 overexpression	17-β-estradiol conversion to potentially genotoxic 16-α-hydroxyestrone. Expression is not associated with the presence of ER and progesterone receptor	[319,342,343]
		Ovarian cancer	CYP3A4, CYP3A5, CYP3A7, and CYP3A43 high protein levels	PXR–CYP3A pathway	[344]
		Prostate cancer	CYP3A5 mRNA and protein levels reduction		[346]
		Endometrial cancer	CYP3A4 expression enhancing	PXR–CYP3A pathway	[355,357]
Diseases Related to the Participation of CYP3A Enzymes in Vitamin D Metabolism		Vitamin D–dependent rickets	CYP3A4 increased activity	Inactivating effect of CYP3A4 on the active form of vitamin D. CYP3A4 I301T mutation.	[361,365,371] [375];
		Osteoporosis	CYP3A4 increased activity	Inactivating effect of CYP3A4 on the active form of vitamin D	[361,365,371]
		Breast cancer, Prostate cancer, Colorectal malignant tumors	CYP3A4 increased activity	Inactivating effect of CYP3A4 on the active form of Vitamin D. Vitamin D takes part in cancer cell proliferation by different ways.	[362,363,384,385,386]
		COVID-19	CYP3A activity	Vitamin D metabolism is catalyzed by CYP3A enzymes	[369]
				Vitamin D attenuates overexpression of inflammatory cytokines	[410]
Inflammation-Dependent Changes in the Expression and Activity of CYP3A Enzymes	Infections	Chronic hepatitis C	Low activity of CYP3A4	Possible CYP3A4 inhibition by IL-6	[396], [398,399].
		HIV	Low activity of CYP3A4		[396], [401]
		COVID-19	Low activity of CYP3A4	CYP3A4 inhibition by IL-6, and TNF	[403]
	Inflammatory Conditions	Type 2 diabetes mellitus	CYP3A suppression	CYP3A4 inhibition by IL-6 and TNF	[382,412]
		Rheumatoid arthritis	Suppression of CYP3A4-mediated metabolism	CYP3A4 inhibition by IL-1Ra, IL-6, and CXCL8	[413] [414] [415,416,417]
		Crohn‘s disease	Significant decrease in protein expression of CYP3A4 in the ileum and colon.		[421]
			Decrease mRNA *CYP3A4* in inflamed small-intestinal tissue		[422]
			Significant decrease of CYP3A proteins in duodenum of children		[423]
		Celiac disease	Marked drop of CYP3A4 activity in vivo		[424]
			Downregulation of mRNA *CYP3A4* in duodenum and ileum		[425]
	Cancer	Advanced ovarian cancer	Reduced CYP3A activity	CYP3A4 inhibition by IL-6, IL-8, and TNF	[427].
		Patients experiencing cancer progression	Significantly low CYP3A4 expression and activity	CYP3A4 inhibition by IL-6	[428,429]
Aberrant Intratumoral Expression of CYP3A Enzymes		Rhabdomyossarcoma	High expression of CYP3A4 and CYP3A5		[434]
		Ewing’s sarcoma metastasis	CYP3A4 overexpression		[436]
		Hepatocellu-lar carcinoma (HCC)	Aberrantly low CYP3A5 expression	CYP3A5 suppresses migration and invasion of HCC cells in vitro via inhibition of ROS–mTORC2–p-AKT signaling.	[438,439].
		Lung adenocarci-noma	CYP3A5 aberrantly underexpression	CYP3A5 significantly reduces the phosphorylation of a TGF-β signaling protein SMAD1, involved in cell growth, apoptosis, development, and immune responses.	[440]
		Prostate cancer	Intratumoral CYP3A5 activation	CYP3A5 mediates the growth of prostate cancer cells by facilitating nuclear translocation of AR.	[442,438]

## Data Availability

Not applicable.

## Abbreviations

3′UTR	3′-untranslated region
AMPKα	AMP-activated protein kinase
AP-1	activating protein-1
AR	androgen receptor
C/EBP	CCAAT/enhancer-binding protein
CAR	constitutive androstane receptor
CCRP	cytoplasmic constitutive active/androstane receptor retention protein
CLEM4	constitutive liver enhancer module 4
COUP-TF	chicken ovalbumin upstream promoter transcription factor
CXCL8	C-X-C motif chemokine ligand 8
CYP	cytochrome P450
DR	direct repeat
EET	epoxyeicosatrienoic acids
ER	estrogen receptor
*ER6*	everted *repeats* 6
FXR	farnesoid X receptor
FXRE	FXR response element
GR	glucocorticoid receptor
HCC	hepatocellular carcinoma
Histone deacetylase 1	histone deacetylase 1
HIV	human immunodeficiency virus
HNF	hepatocyte nuclear factor
HNF4A-AS1	hepatocyte nuclear factor 4α antisense RNA 1
HSP90	heat shock protein 90
IL	interleukin
JAK	Janus kinase
LAP	liver-enriched transcriptional activator protein
LCA	lithocholic acid
LIP	liver-enriched transcriptional inhibitory protein
lncRNA	long noncoding RNA
LUAD	lung adenocarcinoma
LXR	liver X receptor
miRNA, miR	microRNA
NF-κB	nuclear factor kappa B
pERK	proline-rich, extensin-like receptor kinase-1
PGC-1α	peroxisome proliferator-activated receptor-gamma coactivator 1 alpha
PPAR	peroxisome proliferator-activated receptor
prP	proximal promoter
PXR	pregnane X receptor
RNA-seq	RNA sequencing
ROS	reactive oxygen species
SHP	small heterodimeric partner
SNP	single-nucleotide polymorphism
*SOCS1*	suppressor of cytokine signaling 1
STAT	signal transducer and activator of transcription
TET	ten-eleven translocation protein
TNF	tumor necrosis factor
USF1	upstream transcription factor 1
VDR	vitamin D receptor
VEGF	vascular endothelial growth factor
XREM	xenobiotic-responsive enhancer module
